# NK cell cytotoxicity is transiently enhanced during acute malaria and modulated by the host microenvironment

**DOI:** 10.1172/jci.insight.198687

**Published:** 2026-04-21

**Authors:** Pengjun Xi, Patrick A. Sandoz, Maximilian Julius Lautenbach, Eleni Bilev, Björn Önfelt, Anna Färnert, Quirin Hammer, Christopher Sundling

**Affiliations:** 1 Division of Infectious Diseases, Department of Medicine Solna, Stockholm, Sweden.; 2 Department of Infectious Diseases and; 3 Center for Molecular Medicine, Karolinska University Hospital, Stockholm, Sweden.; 4 Department of Applied Physics, Science for Life Laboratory, KTH Royal Institute of Technology, Stockholm, Sweden.; 5 Department of Materials Science and Engineering, Science for Life Laboratory, Uppsala University, Uppsala, Sweden.; 6 Center for Infectious Medicine, Department of Medicine Huddinge, Karolinska Institutet, Stockholm, Sweden.; 7 Institute of Immunology, Christian-Albrecht-University of Kiel, Kiel, Germany.

**Keywords:** Immunology, Infectious disease, Inflammation, Cytokines, Malaria, NK cells

## Abstract

Natural killer (NK) cells are pivotal in the early immune response to *Plasmodium falciparum* infection, yet their functional dynamics and regulation remain incompletely understood. In a longitudinal study of patients with malaria in a nonendemic setting, we observed a transient but potent activation of NK cell cytotoxicity during acute malaria, characterized by rapid granzyme B–mediated killing and elevated expression of genes associated with cytotoxicity (*PRF1*, *GZMB*, and *GZMA*). This heightened activity was supported by increased plasma levels of granzymes and proinflammatory cytokines, which enhanced NK cell function in vitro. However, plasma samples from clinical malaria also contained inhibitory mediators, including soluble cytokine receptors, which dampened NK cell responses. These findings reveal that the host microenvironment orchestrates a tightly regulated NK cell response that potentiates cytotoxicity during acute infection and rapidly downmodulates it after treatment. Understanding this balance between activation and suppression may inform strategies to harness NK cells for malaria control while minimizing immunopathology.

## Abstract 2

Xi et al. show how NK cells transiently enhance cytotoxic functions during acute malaria controlled by the host microenvironment, followed by expansion of NK memory

## Graphical abstract

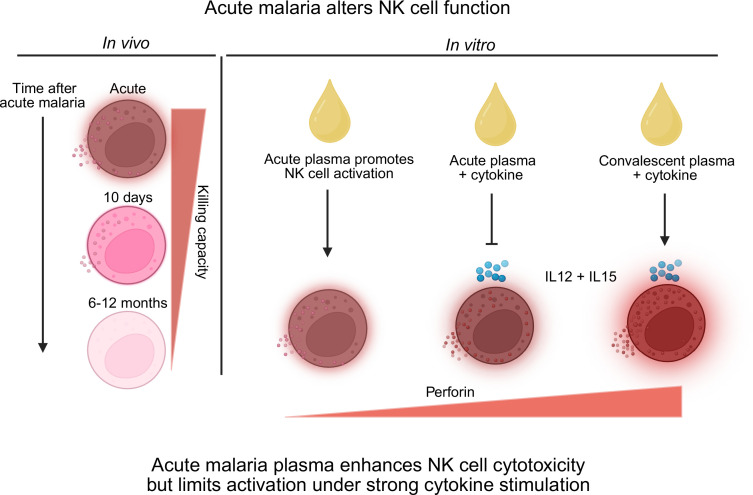

## Introduction

Malaria, caused by protozoan parasites of the *Plasmodium* genus, remains a major global health challenge, particularly in endemic regions such as sub-Saharan Africa and Southeast Asia. Among the *Plasmodium* species, *P*. *falciparum* is the most virulent, responsible for the majority of malaria-related morbidity and mortality (1). According to the latest World Malaria Report from the World Health Organization (WHO), there were an estimated 282 million malaria cases and approximately 610,000 malaria-related deaths globally in 2024 (2).

The immune system plays a crucial role in defending the host against malaria parasites by preventing infections, eliminating parasites, limiting symptoms, and establishing long-term immunity (3). While adaptive immunity is essential for protection, the early innate immune response is crucial for limiting parasite replication (3). Among immune cells, natural killer (NK) cells act as one of the first responders to malaria infection by producing IFN-γ in response to infected RBCs (iRBCs) during the blood stage of the infection (4, 5). In addition, NK cells exhibits increased antibody-dependent degranulation, reflected by CD107a (6), and adaptive-like NK cell expansion, associated with protection from malaria (7).

NK cells are heterogeneous and commonly classified based on the expression of CD56 and CD16, and more recently in the context of malaria with Fc receptor γ-chain (FcRγ) and the transcription factor promyelocytic leukemia zinc figure (PLZF) (6, 7). CD56^bright^ NK cells express higher levels of cytokine receptors, making them more responsive to endogenous cytokines (8). Sensing proinflammatory cytokines triggers CD56^bright^ NK cells to secrete their own cytokines. In contrast, CD56^dim^CD16^+^ have enhanced cytotoxic capacity through abundant expression of the cytotoxic molecules perforin and several different granzymes (9). Moreover, CD56^dim^CD16^+^ NK cells express FcγRIIIα (CD16α), a low-affinity receptor to the Fc domain of specific antibody-antigen complex, enabling antibody-dependent cellular cytotoxicity (ADCC) via immunological synapse formation (9). Additionally, a small subset of CD56^–^CD16^+^ NK cells, initially considered dysfunctional (10), have been shown to expand during chronic or repeated infections and exhibited enhanced ADCC activity compared with CD56^dim^ NK cells (7).

FcRγ and PLZF, expressed by the genes *FCER1G* and *ZBTB16*, respectively, are variably expressed by NK cell subsets. FcRγ can be expressed by both CD56^bright^ and a proportion of CD56^dim^ NK cells, while PLZF is almost exclusively expressed by a proportion of CD56^dim^ cells (6). Low expression of PLZF and FcRγ is associated with NK cell memory (or adaptive NK cells) in viral infection (9). In malaria, a larger proportion of PLZF^–^ and FcRγ^–^ cells is associated with greater ADCC activity and protection against symptomatic disease (6, 7, 11). The enhanced ADCC activity was also associated with previously malaria-exposed compared with naive individuals and with reduced IFN-γ responses (12).

Unlike B cells and T cells, NK cells do not require specific antigen recognition for activation (13), although it was recently shown that repetitive interspersed families of polypeptides (RIFINs) from malaria parasites could provide activating and inhibitory signals to NK cells directly (14). NK cell activation is regulated through 3 complementary pathways: cytokine stimulation, ADCC, and a balance of activating versus inhibitory receptor signals (5). NK cells can be potently activated in vitro by cytokines such as IL-2, IL-12, IL-15, and IL-18 (5), with different combinations resulting in varying degrees and differing quality of activation (15). Cytokine-activated NK cells can acquire memory-like properties, showing enhanced responses upon restimulation (16, 17). Such memory-like NK cell responses have been observed upon ex vivo restimulation of NK cells from individuals in malaria endemic regions with iRBCs (6, 7). Receptors mediating NK cell activation include, for example, NKG2C and NKG2D, while NKG2A is associated with inhibition (18). NKG2C and NKG2A both form heterodimers with CD94 and bind to HLA-E. Coexpression of NKG2C and NKG2A has been associated with elite controllers in HIV infection (19), while CMV infection is associated with expanded CD57^+^NKG2C^+^ NK cells and less NKG2A^+^ cells (20, 21). In individuals living in malaria endemic areas of Mali; however, NKG2C^+^ NK cells were not associated with parasite control (6), potentially indicating pathogen-specific NK cell regulation.

During the blood stage of malaria, asexual parasite replication drives immune activation and clinical symptoms (3). NK cells typically kill target cells via perforin- and granzyme-mediated cytotoxicity or more slowly via death receptor pathways. Evidence from γδ T cell studies demonstrates that granulysin and granzyme B, together with perforin, are critical for killing iRBCs (22), while similar direct evidence for NK cells is lacking. Early IFN-γ production by NK cells, and possibly γδ T cells, in the presence of myeloid cells and supplemented with IL-12 and IL-18, is associated with parasite clearance and the development of adaptive immunity (23). IFN-γ also enhances macrophage-mediated phagocytosis, a mechanism broadly relevant to host defense (24), although it remains unclear if it directly contributes to controlling parasitemia. Monocytes have been shown to work in concert with NK cells to clear parasite material from NK-lysed iRBCs in the presence of parasite-specific antibodies (25), potentially indicating a role in clearing dead parasite material from circulation. In a mouse model of malaria infection, IL-12 deficiency impairs IFN-γ production by NK cells, leading to significantly higher parasitemia at the acute stage of infection as well as recurring parasitemia during chronic infection compared with WT mice (26).

Despite growing evidence for the role of NK cells in malaria, most studies have been cross-sectional or conducted in endemic settings where repeated exposure complicates interpretation. Here, we leverage a longitudinal cohort in a malaria-free setting to characterize NK cell responses over 1 year following acute *P*. *falciparum* infection. We identified a potent but transient activation of NK cell effector functions during acute malaria modulated through the interplay of stimulatory and inhibitory proteins in the microenvironment.

## Results

### Time-dependent kinetics of NK cell subsets and protein expression in response to malaria infection.

**Figure 1:**
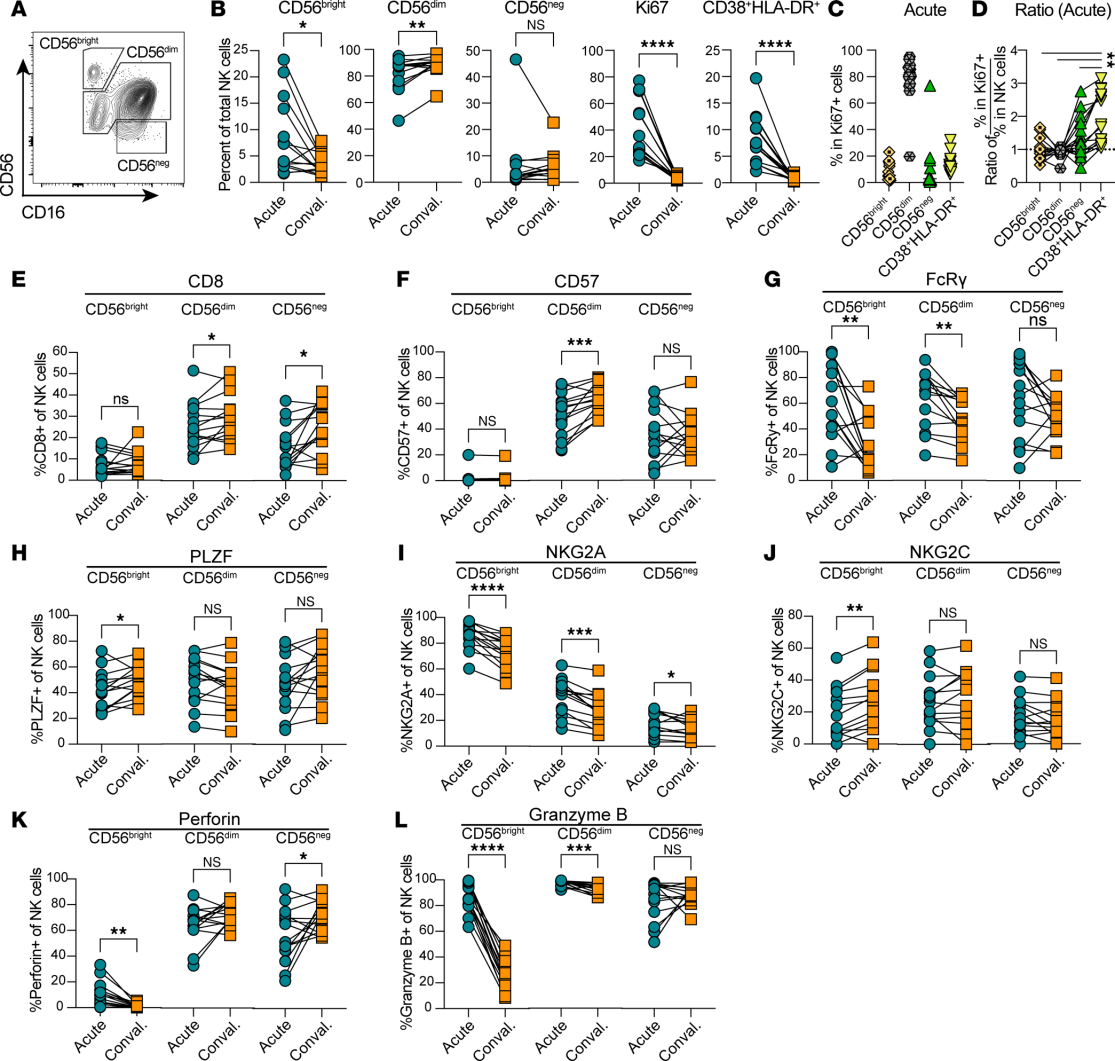
NK cell dynamics and protein expression during and after acute malaria. (**A**) Representative FACS plot showing gating strategy for NK cell subsets based on CD56 and CD16 expression. (**B**) Percent CD56^bright^, CD56^dim^, CD56^–^, proliferating (Ki67^+^), and activated (CD38^+^HLA-DR^+^) cells out of total NK cells. (**C**) Percent Ki67^+^ cells among NK cell subsets and activated NK cells. (**D**) The ratio between Ki67^+^ cells out of total NK cells, indicating preferential enrichment. (**E–L**) Expression of NK cell markers (**E**) CD8, (**F**) CD57, (**G**) FcRγ, (**H**) PLZF, (**I**) NKG2A, (**J**) NKG2C, (**K**) perforin, and (**L**) granzyme B among CD56^bright^, CD56^dim^, and CD56^–^ NK cells. Protein levels were compared between acute (blue circles) and convalescent (12 month; orange boxes) time points for matched donor samples (*n* = 14). Statistical analyses for (**B** and **E**–**L**) were done using 2-tailed paired *t* tests. Analysis for (**D**) was done using one-way ANOVA followed by Tukey’s post hoc test. *P < 0.05, **P < 0.01, ***P < 0.001, ****P < 0.0001.

NK cells were assessed by spectral flow cytometry during acute symptomatic malaria and at convalescence (12 months after treatment) (*n* = 14 matched donors). NK cell subsets were gated for CD56^bright^, CD56^dim^, and CD56^–^ cells (Figure 1A). To understand functional implications, we measured several proteins on the cell surface (CD8, CD57, NKG2A, and NKG2C) and intracellular (FcRγ, PLZF, perforin, and granzyme B) proteins (Supplemental Figure 1, A–C; supplemental material available online with this article; https://doi.org/10.1172/jci.insight.198687DS1).

The frequency of CD56^bright^ NK cells was significantly increased during acute malaria compared with convalescence, while the frequency of CD56^dim^ cells was reduced (Figure 1B). This was primarily due to reduced counts of CD56^dim^ NK cells during acute infection (Supplemental Figure 1D). The frequency and count of proliferating (Ki67^+^) and activated NK cells (CD38^+^HLA-DR^+^) was significantly higher during acute malaria compared with convalescence, with an average Ki67^+^ frequency of 42% versus 3.2% and CD38^+^HLA-DR^+^ frequency of 8.1% versus 0.8%, respectively (Figure 1B and Supplemental Figure 1E). Among the proliferating cells, CD56^dim^ cells were most abundant (Figure 1C). However, calculating proportions between proliferating and total NK cells, only activated (CD38^+^HLA-DR^+^) NK cells were specifically enriched among proliferating cells (ratio > 1), indicating an overall broad proliferation of NK cells during acute malaria (Figure 1D).

Investigating changes in protein levels, significantly more CD56^bright^ NK cells expressed FcRγ, NKG2A, perforin, and granzyme B and less PLZF and NKG2C during acute malaria compared with convalescence (Figure 1, E–L). Similarly, more CD56^dim^ NK cells expressed FcRγ, NKG2A, and granzyme B during acute malaria compared with the convalescent time point. In contrast, cells expressing CD8 and CD57 levels significantly more frequent at convalescence (Figure 1, E–L). Significantly more CD56^–^ cells expressed NKG2A during acute malaria, but fewer CD8 and perforin.

Repeated malaria exposure is known to reshape the immune landscape, but its long-term effect on NK cell profiles remains unclear, especially in the context of reexposure. To investigate this, we reanalyzed an NK cell dataset from a previously published immune landscape study, which included travelers with acute malaria who were infected for the first time (*n* = 24) and individuals who had grown up in endemic areas and had reported a previous history of malaria exposure but had lived in nonendemic area for an average of 13.4 years (*n* = 48) (Supplemental Table 1) (27). NK cells were gated based on CD56 and CD16, and total NK cell numbers and subset dynamics were assessed over 1 year after treatment (Supplemental Figure 2). We observed overall higher counts of total NK cells and of several NK cell subsets for previously exposed donors compared with primary infected individuals. However, this effect was not clear when adjusting for time after diagnosis. There was a clear overarching time-dependent kinetics, both associated with initial lymphopenia but also with subset-specific regulation over the 1 year of follow-up. Consistent with Figure 1F, CD57^+^ NK cells increased over time in both datasets (Supplemental Figure 2C).

In summary, longitudinal NK cell phenotyping revealed both kinetic and functional changes among NK cell subsets following acute malaria. These findings suggest that the acute infection is a strong driver of NK cell remodeling, while immune history older than 13 years had a limited effect in this setting. This could indicate that NK cell memory has a shorter half-life as compared with γδT cells, atypical B cells, and antibody responses characterized in the same cohort (27–30), where prior malaria exposure had a significant effect.

### Acute malaria primes NK cells to kill targets using granzyme B.

**Figure 2:**
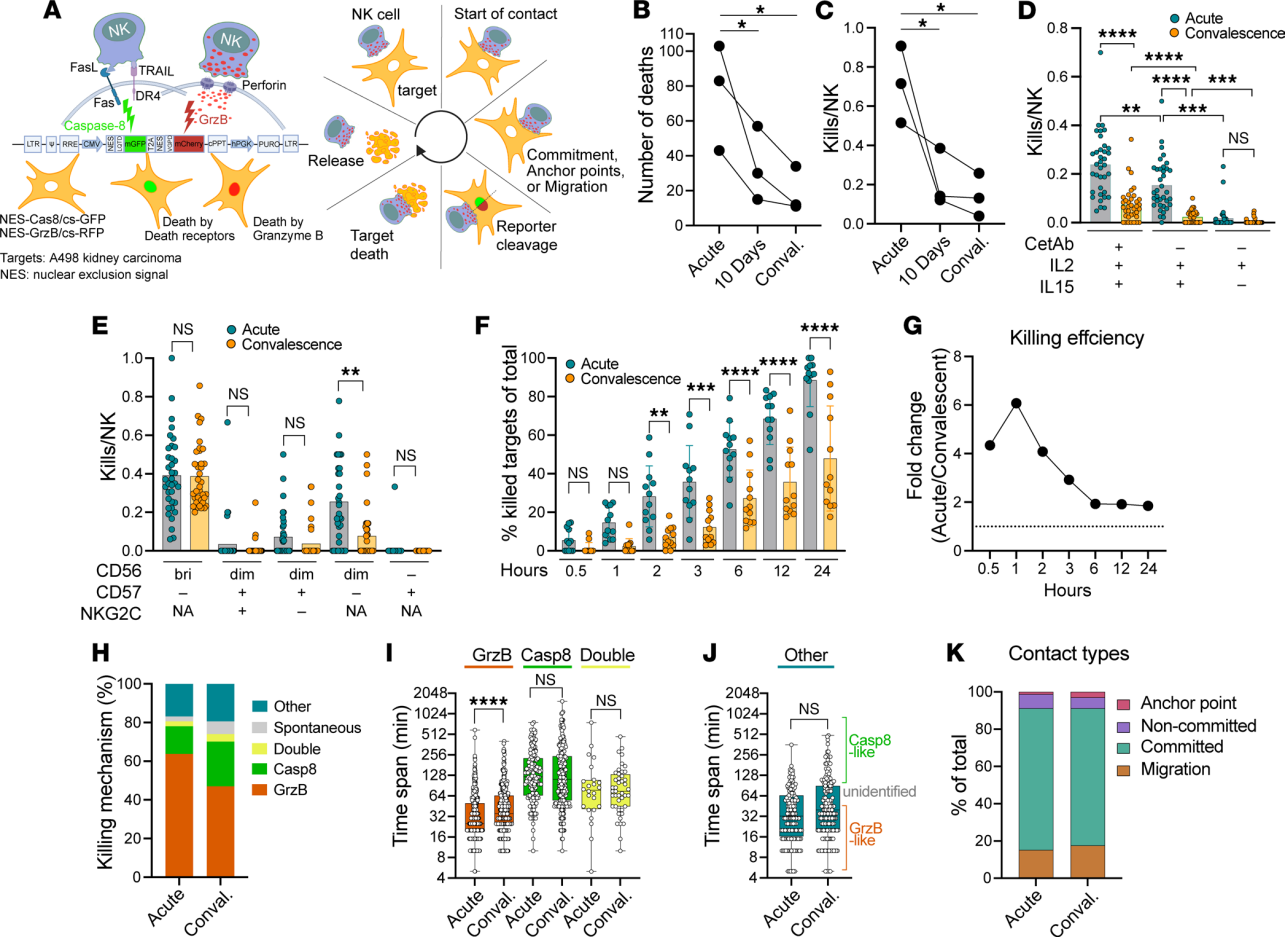
NK cells have increased killing capacity during acute malaria infection. (**A**) Schematic overview of NK cell killing mechanisms and contact types assessed using A498^GBDR^ dual-reporter target cells. (**B**) Total killing by CD56^+^ NK cells from acute, day 10, and convalescent (12-month) time points. Each line represents a patient with malaria. (**C**) Killing efficiency normalized to the number of NK cells per microwell. (**D**) Killing capacity of CD56^+^ NK cells under different stimulation conditions (IL-2, IL-15, and/or CetAb antibody). (**E**) Killing efficiency of sorted NK cell subsets from acute and convalescent time points, stimulated with IL-2, IL-15, and CetAb antibody. NA indicates that the marker was not assessed during cell sorting. (**F**) Time-resolved killing efficiency of CD56^+^ NK cells over the first 24 hours of coculture. Each dot represents a microwell (*n* = 35–37 microwells) in **D**–**F**. (**G**) Ratio of killing efficiency, shown in **F**, between acute and convalescent NK cells over time. Each dot represents the ratio at a given time point. (**H**) Distribution of killing mechanisms used by CD56^+^ NK cells. (**I**) Time from NK cell–target cell contacts to target cell lysis for granzyme B– and caspase-8–mediated killing (*n* = 93–595 killing events). (**J**) Killing time for events classified as “other,” including atypically fast or slow killing events (*n* = 119 and 158 killing events). (**K**) Contact types used during granzyme B–mediated killing by CD56^+^ NK One-way ANOVA followed by 2-tailed paired (**B** and **C**) or 2-tailed unpaired (**D**–**F** and **I**) *t* tests with correction for multiple comparisons; unpaired *t* test for **J**. *P < 0.05, **P < 0.01, ***P < 0.001, ****P < 0.0001. (**H**–**K**) One representative donor based on 36 microwells, see Supplemental Figure 4 for the second donor.

To assess whether NK cell cytotoxic function mirrored the observed kinetic changes over time, we performed time-lapse confocal microscopy in a microwell chip using a dual-reporter A498 kidney carcinoma cell line (A498^GBDR^) that allows us to distinguish between granzyme B– and death receptor–mediated killing (Figure 2A) (31, 32). This system enabled real-time tracking of NK cell killing strategies, timing, and contact dynamics at single-cell resolution (Figure 2A); Supplemental Figure 3, A–C; and Supplemental Videos 1–3, showing killing mechanisms and contact types).

We first compared CD56^+^ NK cells sorted from acute, day 10, and 12-month (convalescent) time points (Supplemental Figure 4A). NK cells from the acute phase exhibited the highest cytotoxicity, while convalescent NK cells showed the lowest (Figure 2B, Supplemental Figure 4B, and Supplemental Figure 3D). This pattern remained consistent when normalizing for NK cell numbers per microwell (Figure 2C). To dissect the contribution of cytokines and antibody-dependent mechanisms, we stimulated NK cells with IL-2 and/or IL-15 and used cetuximab (CetAb) to coat target cells with IgG1 antibody (Ab). Killing was minimal without stimulation but markedly enhanced by IL-15 and CetAb (Figure 2D and Supplemental Figure 4B). IL-15 synergized with IL-2 to boost cytotoxicity, and CetAb further amplified this effect. Notably, acute NK cells consistently outperformed convalescent NK cells under all stimulation conditions (Figure 2D and Supplemental Figure 4B).

We next evaluated cytotoxicity across NK subpopulations sorted by CD56, CD57, and NKG2C expression without using CD16, as that could influence antibody-dependent functions (Supplemental Figure 4A). CD56^dim^CD57^–^ NK cells showed the highest killing capacity while the CD57^+^ memory-like NK cells were less cytotoxic (Figure 2E). However, subset-specific killing patterns were inconsistent across replicates (Supplemental Figure 4C), while it remained consistent for the total CD56^+^ NK cells, suggesting that overall cytotoxicity differences were not driven by a single subset. This is also consistent with our NK cell phenotyping data indicting granzyme B upregulation among both CD56^bright^ and CD56^dim^ NK cells during acute malaria.

We then analyzed the kinetics of target cell killing. Acute NK cells initiated killing more rapidly and achieved higher cumulative cytotoxicity within the first 24 hours compared with convalescent NK cells (Figure 2F). The ratio of acute/convalescent killing efficiency peaked at 1–2 hours with a 4- to 15-fold difference, which then declined over time (Figure 2G and Supplemental Figure 4E), consistent with a shift from rapid granzyme B–mediated killing to slower death receptor–mediated mechanisms (33).

Analysis of killing strategies revealed that granzyme B–mediated cytotoxicity dominated (>50%) in both acute and convalescent NK cells, followed by caspase-8–mediated killing (~20%) and other or unclassified mechanisms (Figure 2H and Supplemental Figure 4F). Importantly, acute NK cells executed granzyme B–mediated killing significantly faster than convalescent NK cells (Figure 2I and Supplemental Figure 4G), while no difference was observed for death receptor–mediated killing. The “other” category included undetermined killing mechanisms that, in some cases, exhibited atypical kinetics (Figure 2J).

Finally, we assessed NK-target cell contact types during granzyme B–mediated killing. Most events involved committed contacts (>2 frames), with no significant differences between acute and convalescent NK cells (Figure 2K and Supplemental Video 3), consistent with the requirement for stable synapse formation in granule-mediated cytotoxicity.

In summary, the enhanced target cell killing by NK cells during acute malaria appears to primarily be driven by increased levels of granzyme B–mediated killing, which were not accompanied by dynamic changes in cell-cell contacts or mediated by specific NK cell subsets.

### NK cells during acute malaria have increased expression of perforin and granzymes but not death ligands.

**Figure 3:**
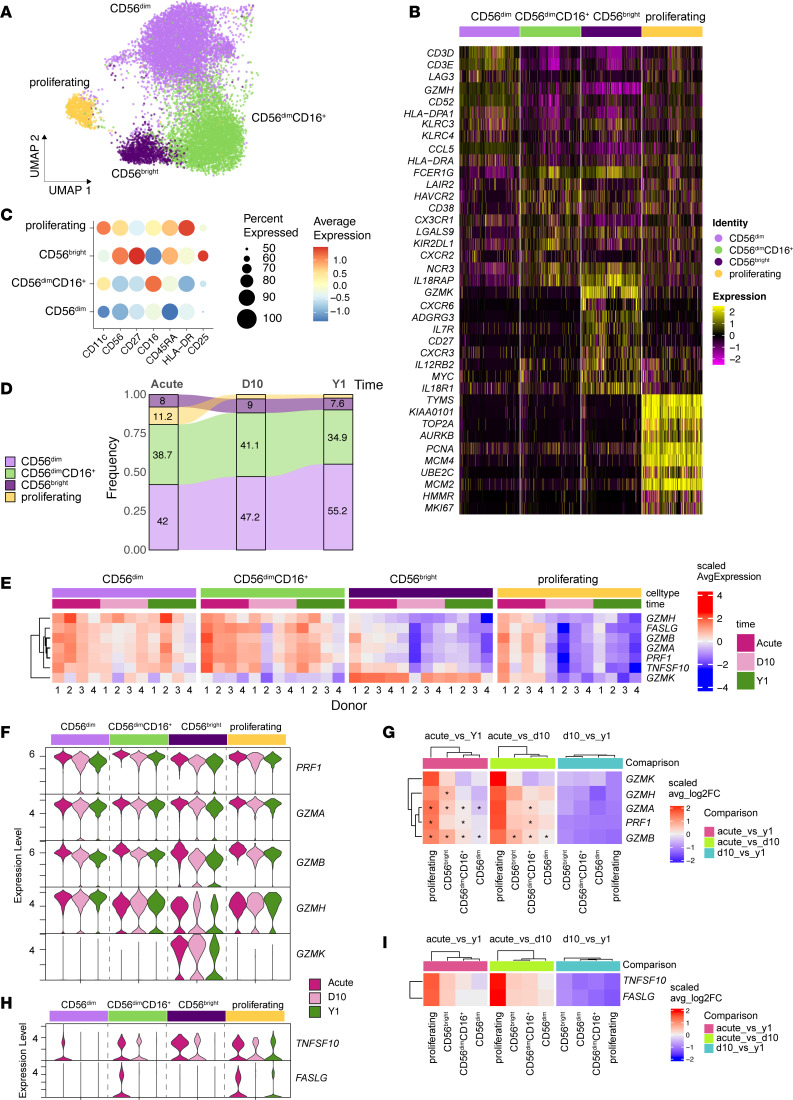
NK cells during acute malaria have increased expression of perforin and granzymes. (**A**) UMAP projection of 12,808 NK cells from 4 patients with malaria across 3 time points (acute, day 10, and 12 months), based on targeted single-cell RNA-seq and antibody-derived surface protein data. (**B**) Heatmap showing scaled expression of key marker genes across the identified NK cell subsets. (**C**) Dot plot of surface protein expression (Ab-Seq) for selected markers used to annotate NK cell subsets. (**D**) Temporal dynamics of NK cell subset frequencies across the 3 time points. (**E**) Individual-specific pseudobulk expression of cytotoxicity-associated (PRF1, GZMA, GZMB, GZMH, GZMK) and death ligand genes (FASLG, and TNFSF10) across NK cell subsets and time points. (**F** and **H**) Violin plots indicating overall expression levels in pooled NK cell subsets. (**G** and **I**) Grouped pseudobulk expression of cytotoxicity associated genes (**G**) and death ligand genes (**I**) across NK cell subsets and time points. Statistical analysis for **G** and **I** was performed using DESeq2 on individual pseudobulk data with *P < 0.01.

To better understand why NK cell cytotoxicity was increased during acute infection compared with later time points, we performed transcriptional analysis of NK cells by repurposing a targeted single-cell multi-omics dataset from 4 donors in the same cohort (34). We extracted NK cells from the dataset, reclustered them (Figure 3A), and annotated 4 major subsets based on gene expression (Figure 3B) and surface protein markers (Figure 3C). The subsets corresponded to CD56^bright^, CD56^dim^, CD56^dim^CD16^+^, and proliferating NK cells (Figure 3, A–C), each displaying distinct transcriptional and surface marker profiles. These largely overlapped with recently described NK2 (CD56^bright^), NK3 (CD56^dim^), and NK1 (CD56^dim^CD16^+^) clusters (35). The CD56^dim^ cells expressed low but detectable levels of CD16 (Figure 3C) and were a proportionally larger population than CD56^dim^CD16^–^ cells shown in Supplemental Figure 2D, suggesting that this cluster included CD16^+^ NK cells with reduced CD16 surface levels. This reduction could suggest recent activation, as also indicated by the increased expression levels of LAG-3 (Figure 3B) and as shown previously in mice (36).

CD56^bright^ NK cells expressed CD27 and CD25 (IL-2R) on the cell surface and high levels of cytokine receptors (e.g., *IL18RAP*, *IL12RB2*, *IL7R*), cytotoxic mediators (*GZMK*), and migration-associated genes (*CXCR3*), consistent with a cytokine-responsive phenotype. CD56^dim^ cells expressed *CCL5* and *GZMH*, suggesting roles in immune recruitment and caspase-independent killing (37). CD56^dim^CD16^+^ cells expressed CD11c on the cell surface and elevated expression of cytotoxicity-associated genes (*NCR3*), cytokine receptors (*IL18RAP*, *CXCR2*), and inhibitory markers (*HAVCR2* [TIM-3] and *LGALS9* [Galectin-9]), indicating a balance of activation and regulation (38). The proliferating NK cells expressed cell cycle genes (e.g., *MCM2*, *MKI67*) and cell surface activation markers (CD11c, HLA-DR), with intermediate CD56/CD16 levels, suggesting a recently activated state and potentially indicating a mix of CD56^bright^ and CD56^dim^ cells (Figure 3, B and C). This is also consistent with the NK phenotyping data (Figure 1C), where all NK subsets could be observed among Ki67^+^ cells.

Temporal analysis revealed that proliferating NK cells were enriched during acute malaria and contracted thereafter, while CD56^dim^CD16^+^ NK cells expanded from acute to day 10, mirroring flow cytometry data (Figure 3D). To link transcriptional changes with cytotoxic function, we assessed pseudobulk expression of *PRF1* (perforin) and granzymes *GZMA*, *GZMB*, *GZMH*, and *GZMK* across subsets and time points at an individual donor level (Figure 3E) and group level (Figure 3, F and G). All NK cell subsets showed significantly elevated *GZMA* and *GZMB* expression during acute malaria compared with 12 months after treatment, while *PRF1* was increased in proliferating and CD56^dim^CD16^+^ cells. *GZMH* was modestly increased in CD56^bright^ cells. *GZMB* was also significantly upregulated for most NK cell subsets compared with 10 days after treatment, while CD56^dim^CD16^+^ cells also expressed significantly more *PRF1* and *GZMA* (Figure 3G). These findings align with the enhanced granzyme-mediated killing observed in vitro. In contrast, expression of death ligands *FASLG* and *TNFSF10* (TRAIL) remained unchanged across subsets and time points (Figure 3, H and I). This suggests that granule-mediated cytotoxicity, not caspase-8–dependent death receptor pathways, dominates during acute malaria.

In summary, acute malaria induces broad transcriptional upregulation of perforin and granzymes across NK cell subsets, supporting a transient but potent cytotoxic phenotype. This transcriptional signature aligns with functional data and underscores the role of granzyme-mediated killing as the primary effector mechanism during acute infection.

### Inflammatory cytokines and granzymes are elevated in plasma during acute malaria.

**Figure 4:**
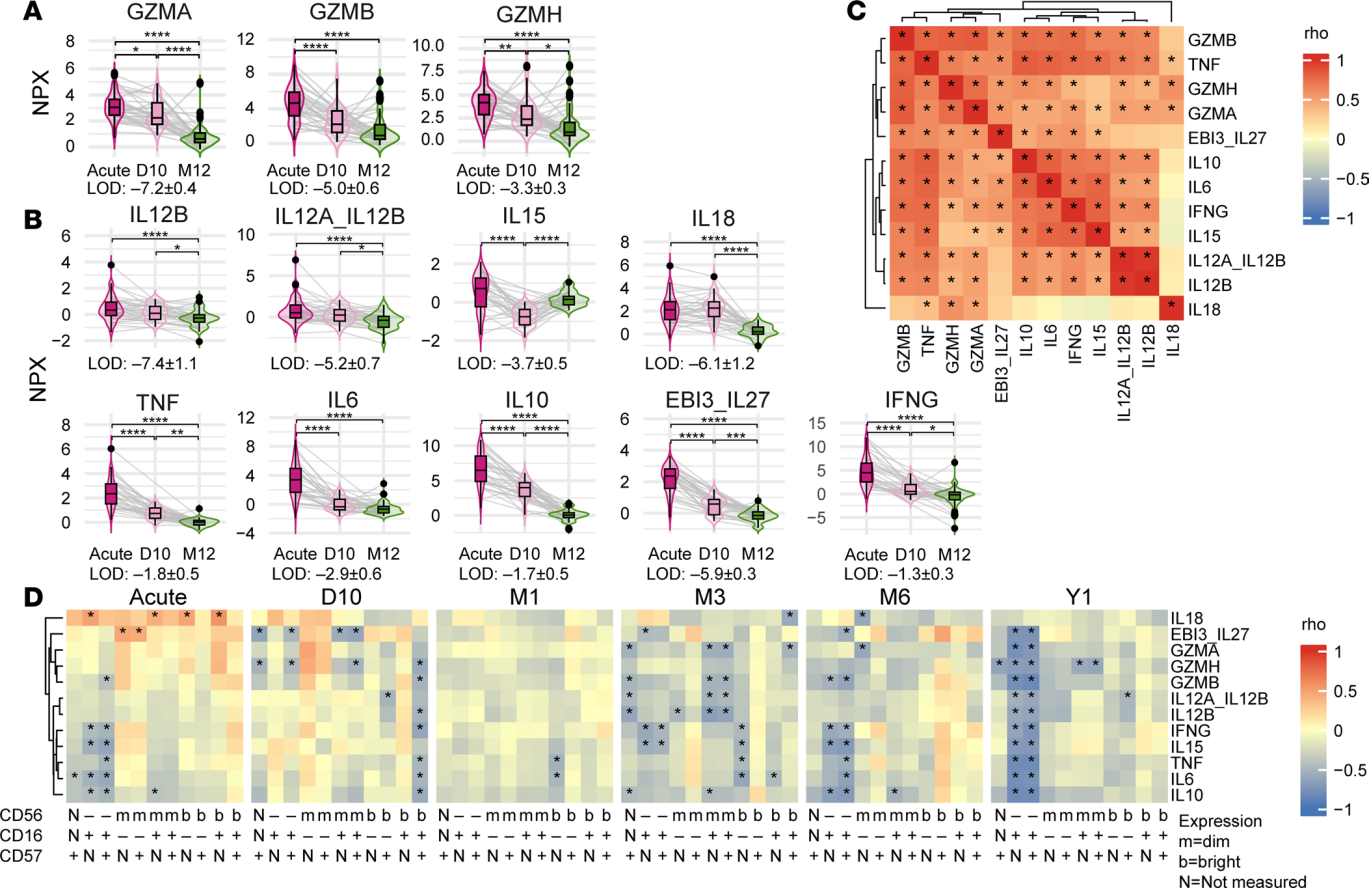
Temporal dynamics of NK cell effector molecule and cytokine abundance. (**A**) Plasma levels of granzymes A, B, and H at acute infection (pink, *n* = 72), 10 days after treatment (light pink, *n* = 27), and 12 months after treatment (green, *n* = 33), measured as normalized protein expression (NPX) were repurposed from Lautenbach et al. (34). (**B**) Plasma concentrations of NK cell–activating cytokines (IL-12, IL-15, IL-18) and general inflammatory cytokines (TNF, IL-6, IL-10, IL-27, IFN-γ) across the same time points from the same dataset. LOD indicates average assay plate limit of detection ± SD for each protein. (**C**) Spearman correlation matrix showing pairwise relationships between cytokines and granzymes at the acute time point. (**D**) Summary of correlations between acute-phase protein levels (ΔNPX from acute to 12 months) and NK cell subset frequencies over time (acute to 12 months) as presented in Supplemental Figure 2. Subsets are defined by CD56, CD16, and CD57 expression. Symbols: (+) = positive expression; (–) = no expression, (b) = bright; (m) = dim; (N) = Not measured. Statistical analyses in **A** and **B** were performed using linear mixed-effects models accounting for repeated measures. *P < 0.05, **P < 0.01, ***P < 0.001, ****P < 0.0001.

Given the enhanced granzyme B–mediated cytotoxicity and transcriptional upregulation of cytotoxic genes in NK cells during acute malaria, we next assessed whether this was reflected in the systemic protein milieu. Using a recently published plasma proteomics dataset from the same cohort (34), we assessed the relative levels of granzymes A, B, and H as surrogate markers of cytotoxic cell activation. All 3 granzymes were significantly elevated during acute malaria and remained higher at day 10 after treatment compared with 12 months (Figure 4A). granzyme B showed the most pronounced increase, consistent with its dominant role in NK cell–mediated killing. These findings support the notion of widespread cytotoxic activity during acute malaria.

We then measured plasma levels of key NK cell–activating cytokines (IL-12, IL-15, and IL-18), which were all significantly elevated during acute malaria (Figure 4B). IL-12 and IL-18 remained elevated at day 10, while IL-15 declined more rapidly. Additional general markers of inflammation (TNF, IL-6, IL-10, IL-27, and IFN-γ) also peaked during acute malaria, indicating a broad inflammatory response. To explore potential coordination among these mediators, we generated a correlation matrix of cytokines and granzymes at the acute time point (Figure 4C). Most proteins were positively correlated, suggesting a coordinated inflammatory program. Notably, IL-18 correlated primarily with GZMA and GZMH, while TNF and GZMB shared similar correlation patterns. IL-10, IL-6, IL-12, IL-15, and IFN-γ formed a larger cocorrelated cluster.

We next examined whether acute-phase cytokine and granzyme levels were associated with NK cell subset dynamics over time. Using Spearman correlations between relative protein levels and NK cell subset frequencies (Supplemental Figure 2), we found that IL-18 and IL-27 were positively associated with multiple NK subsets during acute malaria. IL-18 was specifically correlated with CD56^–^CD16^+^, CD56^bright^CD16^+/–^, and CD56^dim^CD16^+^ cells while IL-27 was correlated with CD56^dim^CD16^–^ NK cells. In contrast, CD56^–^ NK cells showed negative correlations with most inflammatory mediators, both during acute malaria, and especially at later time points (Figure 4D). This indicates that the levels of CD56^–^ NK cells are associated with reduced inflammation and potentially milder disease.

In summary, acute malaria is characterized by a surge in plasma granzymes and proinflammatory cytokines, which are coregulated and correlate with specific NK cell subsets. These findings reinforce the role of the inflammatory microenvironment in shaping NK cell activation and function during acute infection.

### IL-15 and IL-12 enhances, while TGF-β suppresses, NK cell cytotoxic potential.

**Figure 5:**
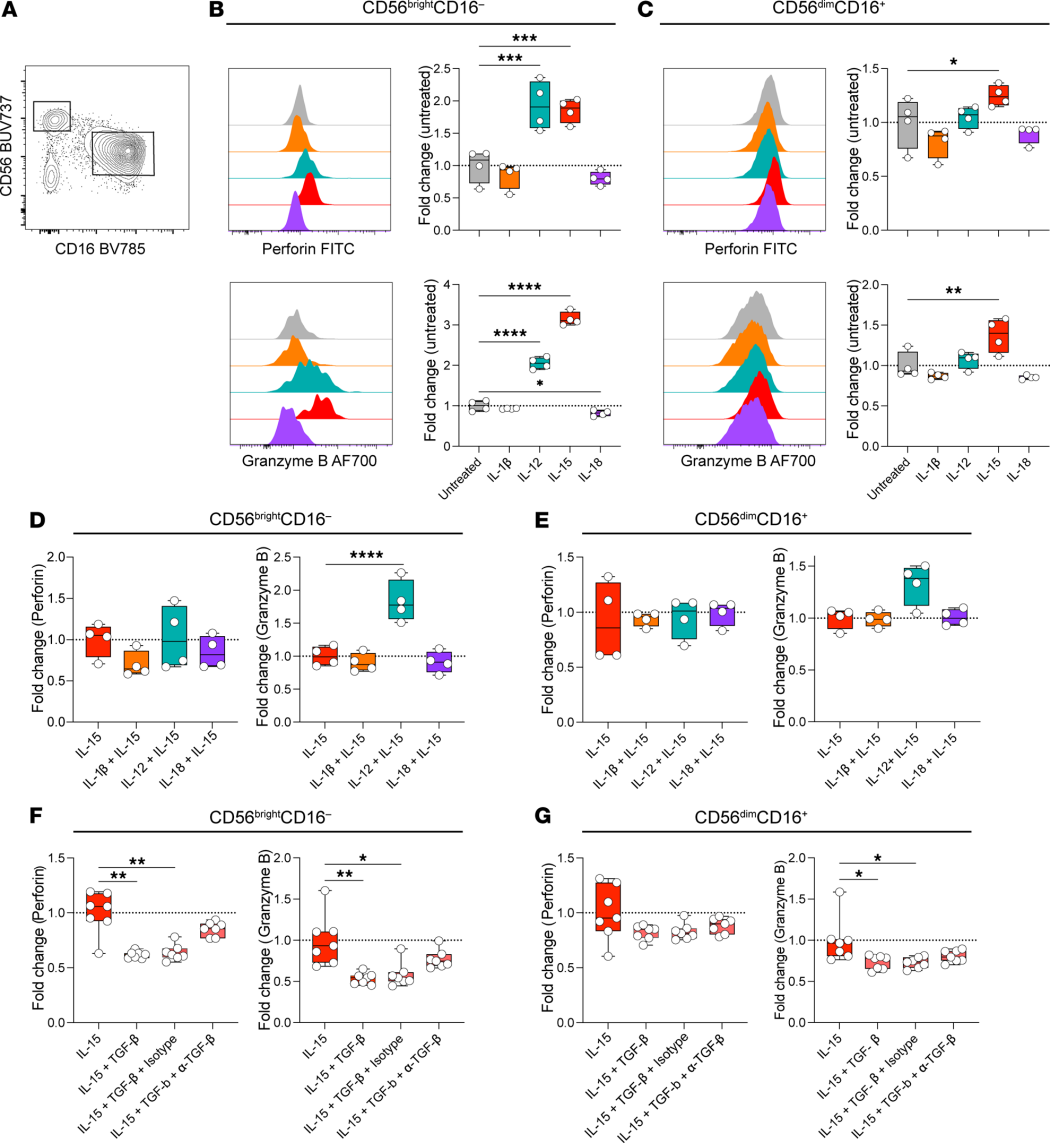
In vitro cytokine stimulation of isolated NK cells from healthy PBMC. (**A**) Representative gating strategy for CD56^bright^CD16^–^ and CD56^dim^CD16^+^ NK cells following 24 hours of in vitro stimulation. (**B** and **C**) Intracellular expression of perforin (top) and granzyme B (bottom) in (**B**) CD56^bright^CD16^–^ and (**C**) CD56^dim^CD16^+^ NK cells following 24h left untreated (gray) or stimulated with IL-1β (orange), IL-12 (turquoise), IL-15 (red), or IL-18 (purple). Values are normalized to the average geoMFI of untreated controls (FBS). (**D** and **E**) perforin (left) and granzyme B (right) expression in (**D**) CD56^bright^CD16^–^ and (**E**) CD56^dim^CD16^+^ NK cells following combined cytokine stimulation. Values are normalized to the average geoMFI IL-15 alone. (**F** and **G**) perforin (left) and granzyme B (right) expression in (**F**) CD56^bright^CD16^–^ and (**G**) CD56^dim^CD16^+^ NK cells after stimulation with IL-15 alone, IL-15 + TGF-β, IL-15 + anti–TGF-β antibody, or IL-15 + isotype control. Values are normalized to the average geoMFI of IL-15 alone. Statistical analyses were done using repeated-measures 2-way ANOVA. *P < 0.05, **P < 0.01, ***P < 0.001, ****P < 0.0001. Results were pooled from 3 separate experiments including in total 4–7 donors.

To investigate how specific cytokines modulate NK cell cytotoxic potential, we stimulated purified NK cells from healthy donors with recombinant cytokines and assessed intracellular levels of perforin and granzyme B in CD56^bright^CD16^–^ and CD56^dim^CD16^+^ NK cell subsets (Figure 5A).

Stimulation with IL-15 significantly increased both perforin and granzyme B in both subsets. IL-12 had a strong effect on CD56^bright^ NK cells but not on CD56^dim^CD16^+^ cells. In contrast, IL-1β and IL-18 alone did not enhance cytotoxic molecule expression in either subset (Figure 5, B and C, and Supplemental Figure 5, A and B). We next tested whether cytokine combinations could synergize to further enhance cytotoxic capacity. Costimulation with IL-12 and IL-15 led to a synergistic increase in granzyme B (but not perforin) in both NK subsets, while combinations with IL-1β or IL-18 did not show additional effects (Figure 5, D and E, and Supplemental Figure 5, C and D).

To assess the effect of immunosuppressive signals, we cultured NK cells with IL-15 alone, IL-15 plus TGF-β, or IL-15 plus TGF-β with or without a blocking antibody. TGF-β significantly reduced perforin and granzyme B levels in both NK subsets, while blockade of TGF-β partly restored cytotoxic molecule expression (Figure 5, F and G, and Supplemental Figure 5, E and F).

In summary, IL-15 and IL-12 act as potent enhancers of NK cell cytotoxic potential, particularly in CD56^bright^ cells, while TGF-β suppresses this enhancement. These findings highlight the dual regulatory role of the cytokine milieu in shaping NK cell effector function during malaria.

### The plasma composition during acute malaria can both stimulate and inhibit NK cell function.

Given the elevated levels of proinflammatory cytokines in acute malaria plasma, we next investigated whether these soluble factors could directly enhance the expression of cytotoxic molecules in NK cells. We cultured sorted NK cells from healthy donors with 20% pooled plasma from either the acute or convalescent (12-month) time points and assessed intracellular perforin and granzyme B levels.

Initial experiments using untreated plasma led to aberrant NK cell phenotypes, including reduced CD56 and CD16 expression and increased cell death (Supplemental Figure 6A). We hypothesized that circulating antibodies in the plasma might be activating or opsonizing NK cells. To address this, we removed IgG using protein G columns, achieving > 99.8% depletion of total and malaria-specific IgG, while preserving IgM (Supplemental Figure 6, B–G). After IgG removal, NK cell viability and phenotype were preserved (Supplemental Figure 6A).

**Figure 6:**
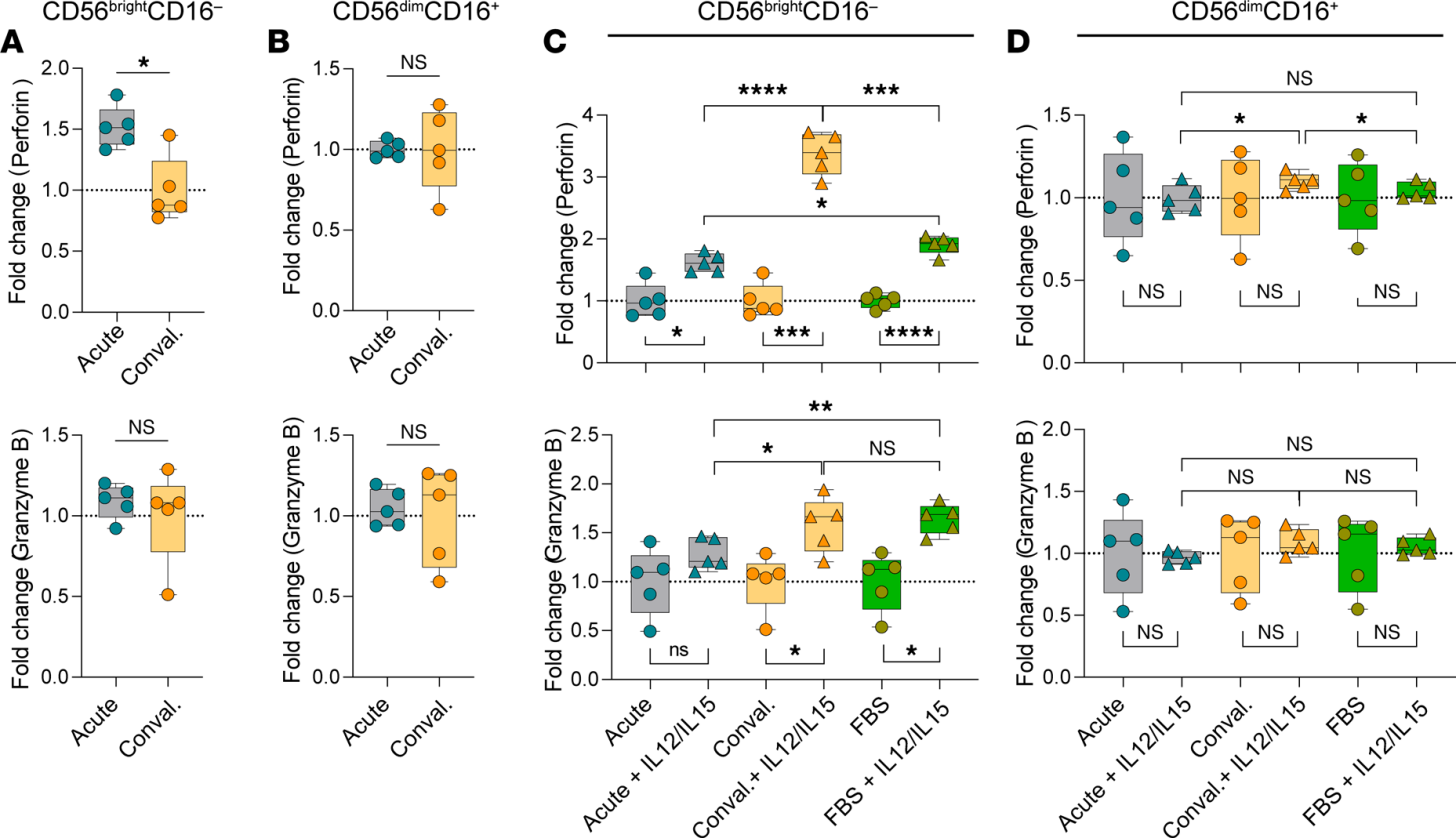
Acute malaria plasma contains both NK cell stimulatory and inhibitory factors. (**A** and **B**) Intracellular expression of perforin (top panel) and granzyme B (bottom panel) in (**A**) CD56^bright^CD16^–^ or (**B**) CD56^dim^CD16^+^ NK cells cultured with 20% IgG-depleted pooled plasma from acute (blue) or convalescent (orange) patients with malaria, respectively. Values are normalized to convalescent plasma-treated controls. (**C** and **D**) Fold change in perforin (top) and granzyme B (bottom) expression in (**C**) CD56^bright^CD16^–^ or (**D**) CD56^dim^CD16^+^ NK cells cultured with pooled plasma or fetal bovine serum (FBS), with or without additional IL-12 and IL-15. Values are normalized to respective average conditions without cytokine supplementation. Statistical analysis was performed using 2-tailed paired Student’s *t* tests. *P < 0.05, **P < 0.01, ***P < 0.001, ****P < 0.0001. Experiments were done on cells sorted from 5 separate donors.

When cultured with IgG-depleted acute plasma, CD56^bright^CD16^–^ NK cells showed significantly increased perforin expression compared with those cultured with convalescent plasma, while granzyme B levels remained unchanged (Figure 6A and Supplemental Figure 7A). No significant changes were observed in CD56^dim^CD16^+^ NK cells (Figure 6B and Supplemental Figure 7B).

To determine whether acute plasma also contained inhibitory mediators, we cultured NK cells with plasma (acute or convalescent) in the presence or absence of exogenous IL-12 and IL-15. In CD56^bright^ NK cells, the addition of cytokines significantly boosted perforin and granzyme B levels, but the enhancement was greater in convalescent plasma than in acute plasma shown both in the calculated ratio (Figure 6C) and geometric mean fluorescent intensity (Supplemental Figure 7C). This suggests that acute plasma contains factors that limit the responsiveness of NK cells to cytokine stimulation. A similar, though less pronounced, pattern was observed in CD56^dim^ NK cells, where only perforin levels were modestly affected (Figure 6D and Supplemental Figure 7D).

In summary, plasma from acute malaria contains both activating cytokines that enhance NK cell cytotoxicity and inhibitory mediators that constrain inflammatory-induced activation. This dual regulation may serve to balance parasite control with the prevention of excessive immune-mediated damage.

### Temporal modulation of pro- and antiinflammatory plasma proteins during and after acute malaria infection.

To better understand how the immune system balances NK cell activation and suppression during malaria, we reanalyzed a comprehensive plasma proteomics dataset (>1,400 proteins) using gene ontology (GO) pathway enrichment analysis (34). We focused on pathways related to immune regulation and NK cell function.

**Figure 7:**
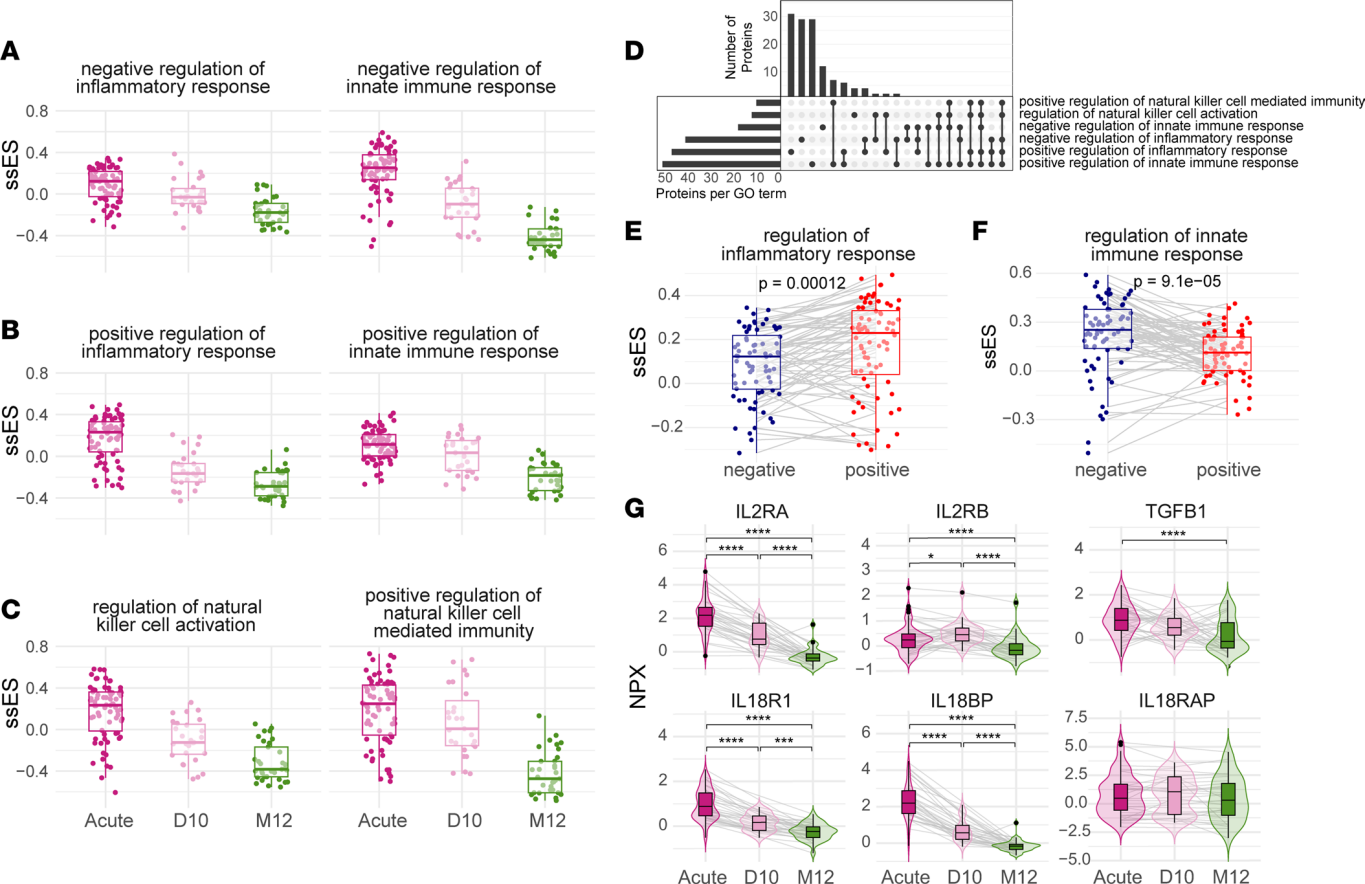
Temporal modulation of NK cell–associated immune regulatory pathways. (**A–C**) Single-sample enrichment scores (ssES) for gene ontology (GO) pathways related to (**A**) negative regulation of immune responses, (**B**) positive regulation of immune responses, and (**C**) NK cell regulation, across acute malaria, day 10 (D10), and 12 months after treatment (M12). (**D**) Upset plot showing the overlap of proteins among the GO pathways analyzed in **A**–**C**. (**E** and **F**) Comparison of ssES for positive (red) and negative (blue) regulation of (**E**) inflammatory response and (**F**) innate immune response pathways at the acute time point. (**G**) Longitudinal plasma levels of selected inhibitory mediators and soluble cytokine receptors relevant to NK cell regulation: TGF-β, IL-2RA (sCD25), IL-2RB, IL-12RB1, IL-18R1, IL-18BP, and IL-18RAP. Protein abundance is shown as normalized protein expression (NPX) at acute (pink, *n* = 72), D10 (light pink, *n* = 27), and M12 (green, *n* = 33) time points. Protein data were repurposed from Lautenbach et al. (34). Statistical analyses were performed using linear mixed-effects models to account for repeated measures and interindividual variability. *P < 0.05, **P < 0.01, ***P < 0.001, ****P < 0.0001.

We first examined positive and negative regulation of immune responses (Figure 7, A and B). Both types of pathways were significantly enriched during acute malaria and declined over time. NK cell–specific regulatory pathways followed a similar pattern, peaking during acute infection and contracting after treatment (Figure 7C). Despite some overlap, most proteins were unique to each pathway (Figure 7D and Supplemental Table 2), indicating distinct regulatory programs.

To assess the balance between immune activation and suppression, we compared enrichment scores for positive versus negative regulation of 2 key processes: the inflammatory response and the innate immune response. During acute malaria, positive regulation dominated the inflammatory response (Figure 7E), suggesting an active proinflammatory state. In contrast, negative regulation was more prominent in the innate immune response (Figure 7F), potentially reflecting a feedback mechanism to limit excessive activation of innate cells, including NK cells.

Because no GO pathway specifically captured NK cell suppression, we directly examined plasma levels of known inhibitory mediators and soluble cytokine receptors that could modulate NK cell activity (Figure 7G). These included TGF-β, soluble IL-2RA (sCD25), IL-2RB, IL-12RB1, IL-18R1, IL-18BP, and IL-18RAP. All except IL-18RAP were significantly elevated during acute malaria, with several remaining elevated at day 10. These proteins may reduce cytokine bioavailability or block receptor signaling, thereby dampening NK cell responses.

In summary, acute malaria triggers a coordinated upregulation of both activating and inhibitory immune pathways. While the inflammatory response is strongly activated, innate immune regulation, including NK cell suppression, is also engaged. This dual modulation likely serves to optimize parasite control while minimizing immunopathology.

## Discussion

NK cells play a critical role in the early immune response to *Plasmodium* infection by rapidly initiating cytotoxic activity and producing cytokines that shape downstream immune responses (39). NK cells contribute to parasite clearance through direct cytotoxicity and ADCC, targeting iRBCs and merozoites (25, 40). In this study, we leveraged a longitudinal cohort in a malaria-free setting to investigate NK cell function and dynamics over 1 year following acute *P*. *falciparum* infection. This unique setting allowed us to examine NK cell responses in individuals with and without prior malaria exposure, free from the confounding effects of reinfection, and to characterize the temporal evolution of NK cell phenotypes and effector functions.

In our cohort, prior malaria exposure did not significantly influence NK cell responses compared with individuals experiencing their first infection. This contrasts with findings from endemic settings, where repeated exposure has been associated with the expansion of adaptive-like NK cell subsets and altered functional profiles (6). A likely explanation for this discrepancy is the long interval since last exposure (on average 13.4 years) before the current infection for previously exposed individuals in our study (27). NK cell memory has been linked to protection from malaria (6) and was shown to persist for several months and up to 5 years in animal models following infection or vaccination if rechallenged by homologous antigen (41). However, persistence beyond a decade has not been demonstrated. Thus, the absence of memory-associated features in our data is consistent with the likely waning of any prior NK cell training. Interestingly, we observed a lower frequency of FcRγ^+^ NK cells at convalescence and delayed expansion of CD57^+^ NK cells after treatment, which may reflect the generation of new memory-like NK cells in response to the acute infection (42).

Repeated malaria infections was shown to drive the expansion of CD56^–^CD16^+^ NK cells in Ugandan children (7) and CD56^dim^ adaptive NK cells in both children and adults in Mali (6). Here, we observed a proportional expansion of CD56^bright^ NK cells during acute infection and a reduction of CD56^dim^ cells compared with the 12-month follow-up. The increase in CD56^bright^ cells was not reflected in the cell counts, however, which pointed toward lymphopenia of especially CD56^dim^ cells changing overall proportions. Furthermore, all NK cell subsets displayed robust proliferation during acute malaria to a proportionally similar extent. This suggests that the inflammatory milieu during acute infection may be a potent driver of overall NK cell expansion and function when compared with redistribution among NK cell subsets. Supporting such a hypothesis, both CD56^bright^ and CD56^dim^ NK cells increased their expression of FcRγ and NKG2A, associated with protective adaptive NK cells during acute infection (6). CD56^bright^ NK cells further significantly upregulated both perforin and granzyme B levels, while CD56^dim^ NK cells increased their already high granzyme B levels, likely enhancing their overall higher cytotoxic activity during acute malaria. These observations of increased NK cell function during infection are in line with those presented by Ty et al. and Hart et al. (6, 7).

The reporter system we employed in this study, originally developed by Liesche et al. (32), enabled us to identify granzyme B–mediated killing as a dominant NK cell effector mechanism during acute malaria. This finding was corroborated by the transient upregulation of *GZMA* and *GZMB* transcripts across NK cell subsets and elevated plasma levels of multiple granzymes, indicating widespread in vivo degranulation. Notably, elevated levels of granzymes were also observed in γδ T cells, activated CD4^+^ and CD8^+^ effector memory T cells (34) during acute malaria, potentially indicating additional sources. Similarly high granzyme levels have not been observed across other febrile infectious diseases (43), suggesting that granzyme-mediated cytotoxicity may represent a particularly prominent trait of acute malaria infection. Elevated granzyme A and B levels were also associated with early immune activation in controlled human malaria infection and clinical malaria in African children (44).

Antibodies are important mediators of NK cell effector functions in malaria infection and vaccination (40). However, NK cell activity is also strongly influenced by the surrounding cytokine milieu, as shown in cancer and other infectious disease contexts (15, 39, 45, 46). In our cohort, cytokines with both positive and negative modulatory functions were elevated during acute malaria, consistent with previous reports in clinical malaria (27). We found that IL-18 and IL-27 levels positively correlated with several NK cell subsets during acute infection, while IL-2, IL-12, and IL-15 enhanced perforin and granzyme B expression, particularly for CD56^bright^ NK cells. This is consistent with these cells expressing more receptors to respond to cytokine stimulation (8) and potentially also because they have low baseline levels of perforin and granzyme prior to activation. These cytokines are also important for sustaining serial target cell killing (47, 48). In support of an improved responsiveness to cytokine stimulation, NK cells sorted from patients with acute malaria were more potent at killing target cells in response to IL-15 than those sorted from convalescent time points. However, translating in vitro cytokine stimulation to the complex in vivo environment remains challenging. When we stimulated purified NK cells with plasma from acute and convalescent time points, acute plasma induced perforin upregulation, consistent with elevated IL-12 and IL-15 levels. However, the addition of exogenous cytokines had a diminished effect in acute plasma compared with convalescent plasma, suggesting the presence of inhibitory factors during acute malaria that constrain NK cell activation.

TGF-β was elevated in plasma during acute malaria and is known to negatively regulate NK cell function, both directly by suppressing cytotoxicity and cytokine production (49) and indirectly by modulating IL-12 production from macrophages and DCs (50). Consistent with this, our data suggest that TGF-β may contribute to the suppression of NK cell activity during acute infection. To further explore the regulatory landscape, we reanalyzed a recently published proteomic dataset from the same cohort, which quantified over 1,400 plasma proteins (34). This allowed us to assess overall immune regulatory pathways and those specifically associated with innate immune responses and NK cell function. We found that both positive and negative immune regulatory pathways were significantly upregulated during acute malaria and contracted following treatment. Notably, positive regulation dominated the overall inflammatory response, while negative regulation was more prominent in pathways associated with innate immunity. This shift may reflect a transition from innate to adaptive immune control and could help explain the rapid downregulation of NK cell cytotoxicity after the acute phase.

We also assessed the levels of several soluble receptors associated with NK cell function that were not included in the GO pathway analysis. All investigated proteins, except IL18RAP, were significantly elevated during acute malaria. These soluble receptors may inhibit cytokine activity by reducing cytokine availability or by blocking receptor-ligand interactions, as has been shown for IL2RA (51), IL18R1 (52), and IL18BP (53). Such mechanisms are thought to play a protective role by limiting excessive inflammation during infection and immune-mediated pathology (51, 54). However, not all soluble receptors are inhibitory. For example, soluble IL-15Rα can enhance the activity of soluble IL-15 to stimulate NK cells and CD8^+^ T cells (55), and its shedding is important for IL-15 trans signaling (56). Together, these findings highlight the complexity of cytokine regulation during malaria and underscore the need for further investigation into how soluble mediators shape NK cell function in both protective and pathological contexts.

In summary, our findings highlight a tightly regulated NK cell response during acute malaria, characterized by rapid cytotoxic activation and subsequent downmodulation. This balance is potentially orchestrated by a complex interplay of activating and inhibitory signals in the host microenvironment, which could be important in contributing to parasite control while limiting immunopathology. These insights advance our understanding of NK cell biology in malaria and may inform future strategies to modulate innate immunity for therapeutic benefit.

### Limitations of the study.

This study offers mechanistic insights into NK cell regulation during acute malaria; however, several limitations should be considered. The reporter cell line, while enabling precise dissection of cytotoxic mechanisms, may not fully recapitulate interactions with infected RBCs. The small donor cohort in single-cell transcriptomics likely reduced sensitivity to detect rare subsets, such as CD56^–^CD16^+^ cells. Furthermore, the transcriptomic data did not include healthy controls, making it difficult to assess potential gene expression changes remaining for > 1 year after the acute malaria infection. Moreover, the long interval since prior malaria exposure, could make the study participants NK cell responses reflect those closer to primary rather than repeated infections.

## Methods

### Sex as a biological variable

Our study examined immune responses during and after malaria using different methodology. The flow cytometric analysis included samples from both males and females. This was also the case for in vitro–stimulated NK cells from healthy donors. The single-cell RNA-seq dataset was based on male samples only to reduce variability considering only 4 donors were investigated.

### Study cohort

The prospective cohort included adult patients diagnosed with *Plasmodium falciparum* malaria at Karolinska University Hospital. Peripheral blood was collected at 6 time points (acute malaria, 10 days, and 1, 3, 6, and 12 months after treatment), and peripheral blood mononuclear cells (PBMCs) and EDTA plasma were isolated and stored separately. Participants were categorized based on malaria exposure history, including primary infected experiencing their first *P*. *falciparum* infection and previously exposed individuals, originating from malaria-endemic regions with self-reported prior infections. Peripheral blood mononuclear cells (PBMCs) were isolated from blood with Ficoll-Paque density gradient separation and resuspended in 90 % fetal bovine serum (FBS) and supplemented with 10% DMSO and stored in liquid nitrogen while plasmas were stored at –80^o^C.

### Data collection

#### Immune cell phenotyping with flow cytometry.

Frozen PBMCs were thawed in a 37°C water bath and diluted 1:1 with cold Iscove’s Modified Dulbecco’s Medium (IMDM) or RPMI supplemented with 2 mM L-glutamine, 100 U/mL penicillin, 100 μg/mL streptomycin, and 10% heat-inactivated FBS. Cells were rested on ice for 20 minutes, washed twice in DPBS (without calcium or magnesium), and stained with Fixable Live/Dead viability dye (Thermo Fisher Scientific) for 20 minutes, followed by additional washing in DPBS supplemented with 2% FBS (ThermoFisher Scientific). The live/dead viability staining was only used for samples comparing primary infected and previously exposed donors (Supplemental Figure 2). The cells were then incubated for 20 minutes on ice, in 2 steps with 2 washes in between, using an antibody mix targeting surface antigens. After staining, the cells were washed twice in DPBS with 2% FBS before acquisition on a 5-laser Cytek Aurora for Figure 1 or a BD LSRFortessa flow cytometer for Supplemental Figure 2 (27). Data were analyzed using FlowJo X (v10.4.2). Cell counts were calculated either as cells per μL using calibright beads (ThermoFisher Scientific) or per 1,000 live lymphocytes.

#### NK cell sorting.

PBMCs were thawed and stained as was done for immune cell phenotyping, including markers for viability and cell lineages (Live/Dead viability Green, CD3, CD14, CD19, CD45, and CD56), to isolate total CD56^+^ NK cells. For subset-specific experiments, additional markers (7-AAD, CD57, and NKG2C) were included. See Supplemental Figure 4 for the gating strategy. Cell sorting was performed using a Sony SH800 cell sorter. Post-sorting, NK cells were rested for 2 hours at 37°C and 5% CO_2_.

### In vitro time-lapse confocal microscopy assay

To assess NK cell cytotoxicity in real time, we used A498^GBDR^ target cells, A498 kidney carcinoma cells stably transduced with the dual fluorescent reporter construct pJML1-LQDT-mGFP-T2A-NES-VGPD-mCherry, as previously described in ref. 31 (provided by Carsten Watzl, Technical University Dortmund, Dortmund, Germany). This system enables discrimination between granzyme B– and caspase-8–mediated killing. Briefly, A498^GBDR^ cells were seeded into 16-compartment 350 μm microwell chips 24 hours prior to adding sorted NK cells. Each microwell compartment was treated with or without 20 ng/mL IL-2, 10 ng/mL IL-15, and 1 μg/mL CetAb at the start of the assay. Imaging was initiated immediately after NK cell seeding and performed using a Zeiss Colibri 7 wide-field microscope equipped with a 10× Plan-Apochromat objective and an environmental chamber maintained at 37°C and 5% CO_2_. Images were acquired every 5 minutes for 40–48 hours.

### Imaging analysis

To quantify NK cell cytotoxicity, time-lapse image sequences were analyzed manually using Zen software (Zeiss, Germany). Each A498^GBDR^ target cell and NK cell was tracked throughout the 48-hour experiment. For every killing event, the following parameters were recorded: time of initial NK–target cell contact, reporter cleavage (indicating granzyme B or caspase-8 activity), time of target cell death, and the inferred killing mechanism. Killing events were classified based on reporter signal dynamics into granzyme B–mediated, caspase-8–mediated, or other/atypical mechanisms. In some cases, image brightness was uniformly adjusted to confirm nuclear relocalization of the fluorescent reporter, aiding in the classification of killing mode.

### NK cell purification and recombinant cytokine stimulation

Frozen PBMCs from healthy donors were thawed and rested for 4 hours in RPMI-1640 medium supplemented with 10% FBS), 2 mM L-glutamine, and 100 U/mL penicillin-streptomycin (all from Thermo Fisher Scientific). NK cells were then isolated using a negative selection kit (Miltenyi Biotec, 130-092-657) according to the manufacturer’s instructions. Purified NK cells (200,000 per well) were seeded into 96-well plates and stimulated for 20–24 hours with recombinant cytokines: IL-15 (0.1–10 ng/mL), IL-12 (10 ng/mL), IL-18 (10 ng/mL), and/or TGF-β (10 ng/mL), anti–TGF-β blocking antibody (10 ng/mL), or IgG1 isotype control antibody (10 ng/mL). Following stimulation, cells were washed with PBS and stained for surface markers for 20 minutes at room temperature in the dark. After washing, cells were fixed with BD Cytofix/Cytoperm for 20 minutes at 4°C, permeabilized with BD Perm/Wash buffer, and stained overnight at 4°C with antibodies against perforin (1:800) and granzyme B (1:400). After final washes, cells were resuspended in FACS buffer (PBS + 2% FBS + 2 mM EDTA, pH 7.4) and acquired on a BD LSRFortessa flow cytometer (BD FACSDiva Software v9.7) and analyzed using FlowJo X.

### Plasma antibody depletion

To avoid the effects of antibodies in pooled plasma, IgG was removed using protein-G columns (Ab SpinTrap, Cytiva) according to the manufacturer’s instructions. Briefly, 100 μL plasma was added to a column containing protein-G and incubated for 10 minutes. They were then centrifuged at 400*g* for 2 minutes to collect the plasma. The IgG bound to the beads was eluted with 600 μL 0.1M glycine-HCl (pH 2.7) twice and neutralized with 25 μL 1M Tris-HCl (pH 9.0). The efficiency of IgG removal from the pooled plasmas was validated with multiplex magnetic bead-based assay and ELISA, further described below.

### NK cell culture with patient plasma

To investigate the effects of soluble factors in malaria plasma on NK cell function, we cultured purified NK cells from healthy donors with pooled plasma from patients with malaria. Plasma was collected either during acute infection (*n* = 53 donors) or at 12 months after treatment (*n* = 26 donors), with equal volume contributions from each donor. NK cells were cultured for 20–24 hours in RPMI-1640 medium supplemented with 20% IgG-depleted plasma (acute or convalescent) or 20% FBS, with or without additional IL-15 (1 ng/mL) and IL-12 (10 ng/mL). After incubation, cells were processed for intracellular staining of perforin and granzyme B.

### Multiplex bead–based assay for pooled plasma IgG

To confirm the efficiency of IgG depletion from pooled plasma, we performed a multiplex bead–based antibody assay targeting total IgG, IgG1, IgG3, and IgM responses against recombinant *P*. *falciparum* merozoite surface antigens of different strains, indicated in the parenthesis. The antigens included AMA1 (3D7 and FVO), MSP1-19 (3D7), MSP2 (DD2 and CH150), MSP3 (3D7 and k1), and GAMA (3D7). Plasma samples (predepletion, postdepletion, and eluted IgG fractions) were diluted 1:100 or 1:1,250 in dilution buffer (1% BSA, 0.05% Tween-20 in PBS, pH 7.4). Antigens were covalently coupled to distinct regions of magnetic COOH beads (Bio-Rad) using the Bio-Plex Amine Coupling Kit, according to the manufacturer’s instructions. Each well received 50 μL of antigen-coupled beads (1,250 beads/region) and 50 μL of diluted plasma and was incubated for 1 hour at room temperature on a shaker in the dark. After 4 washes with PBS + 0.05% Tween-20, beads were incubated with PE-conjugated secondary antibodies specific for total IgG (Jackson ImmunoResearch) or IgG1, IgG3, or IgM (Southern Biotech) for 30 minutes. Following additional washes, samples were resuspended in dilution buffer and acquired on a Bio-Plex 200 system (Bio-Rad), with a minimum of 100 beads per region analyzed. Median fluorescence intensity (MFI) was used to quantify antibody levels.

### ELISA for pooled plasma IgG

To further validate IgG depletion from pooled plasma, we performed an ELISA targeting total human IgG. High-binding 96-well plates (Costar) were coated overnight at 4°C with goat anti–human IgG (Merck, I2136) at 1 μg/mL in PBS (30 μL/well). After washing 4 times with PBS + 0.05% Tween-20, wells were blocked with 100 μL of dilution buffer (1% BSA, 0.05% Tween-20 in PBS, pH 7.4) for 1 hour at room temperature. Plasma samples (predepletion, postdepletion, and eluted IgG fractions) were serially diluted (1:100 starting dilution, 5-fold series, 8 dilutions total) and added in duplicate (100 μL/well). Plates were incubated for 30 minutes at room temperature in the dark. Following 4 washes, wells were incubated with 100 μL of peroxidase-conjugated goat anti–human IgG (Jackson ImmunoResearch) diluted 1:10,000 in dilution buffer for 30 minutes. After a final 4 washes, 75 μL of TMB substrate (Thermo Scientific) was added and incubated for 5–10 minutes until color developed. The reaction was stopped with 75 μL of 0.2M H_2_SO_4_, and absorbance was measured at 450 nm using a spectrophotometric plate reader (Thermo Scientific).

### Single-cell multi-omics data set

We analyzed previously published targeted single-cell multi-omics data generated from PBMCs of malaria-infected individuals enrolled in the Swedish cohort (34). Samples from 4 individuals (2 primary-infected, 2 previously exposed) were collected at 3 time points: acute infection, day 10, and 12 months after treatment. CD45^+^ cells were profiled using the BD Rhapsody system with the Immune Response Panel (targeting 399 genes) and 29 oligo-conjugated Ab-Seq antibodies (BD Biosciences). A total of 73,121 cells were annotated into 27 immune cell subsets based on the expression of 374 genes and 29 surface markers, as previously described (34). For this study, NK cells were digitally isolated, reintegrated, and reclustered to remove potential contaminating populations. All downstream analyses were performed using the Seurat R package (v4) (57).

To account for interindividual variability and avoid single-cell level biases, we performed pseudobulk differential expression analysis. For each donor and time point, raw counts from all NK cells were aggregated to create donor-level pseudobulk profiles using Seurat’s AggregateExpression(). These aggregated counts were then normalized and analyzed using Seurat’s function FindMarkers() with DESeq2 (v1.40) to identify genes significantly differentially expressed between acute infection and subsequent time points. The design formula included the donor as a blocking factor to control for individual-specific effects. Genes with a *P* < 0.01 were considered significant.

### Differential protein abundance analysis

Plasma protein data published previously by Lautenbach et al. (34) contained measurements of proteins during and after acute *P*. *falciparum* malaria from the same cohort of returning travelers. We used these data to perform differential abundance protein analysis to compare the abundance levels between different groups at various time points. The analysis was conducted using a linear mixed-effects model with the formula.

NPX ~ time point + (1 | subject), accounting for random effects due to subjects using the lmerTest in R (58). Post hoc pairwise comparisons were conducted using the emmeans package to identify significant differences between sampling time points (59). Statistical significance in figures is denoted as *P ≤ 0.05, **P < 0.01, ***P < 0.001, ****P < 0.0001

### Correlation analysis

To explore associations between circulating protein levels and NK cell subset dynamics, we performed Spearman correlation analyses using the correlation package in R (60). Changes in protein abundance were quantified as _Δ_NPX, representing within-individual differences between the acute and 12-month time points. NK cell subset frequencies were derived from flow cytometry data from ref. 27 and matched to protein data for overlapping time points (*n* = 42 individuals with complete paired data. Correlation analyses were restricted to these paired samples to ensure robust comparisons across modalities.

### GO pathway analysis

To evaluate functional changes in circulating proteins, we performed pathway-level analysis using GO terms related to biological processes (61). Protein sets corresponding to selected GO terms were identified based on overlap with the Olink Explore 1536 panel. Sample-wise enrichment scores were calculated using gene set variation analysis (GSVA), implemented via the GSVA package in R (62). This unsupervised method enabled quantification of pathway activity across individual samples without requiring predefined groupings. Enrichment scores were used to assess temporal dynamics of immune regulatory pathways, including those related to NK cell function, inflammation, and innate immune responses.

### Statistics

Statistical analyses were performed using GraphPad prism version 11 or R and are further described in the figure legends. Briefly, analyses comparing donor responses or characteristics over time or following multiple stimulations were done using repeated measures 2-tailed 1-way or 2-way ANOVA. If the analyses included missing data, a linear mixed effects model was used instead. P-values < 0.05 were considered significant with *P < 0.05, ** *P* < 0.01, ***P < 0.001, and ****P < 0.0001.

### Study approval

The study was conducted at Karolinska University Hospital in Stockholm, Sweden. All participants provided written informed consent. The prospective malaria cohort work was approved by the Ethical Review Board in Stockholm and the Swedish Ethical Review Authority under the following permits: Dnr 2006/893-31/4, 2013/550-32, 2015/2200-32, 2016/1940/32, 2018/2354-32, 2019-03436, 2020-00859, 2020-04147, and 2024-00374-02. The work with NK cells from healthy human blood was approved under permit Dnr 2025-01197-01.

### Data availability

European Data Regulations preclude open deposition of sensitive personal data into public repositories. This includes biological data that can be traced back to an individual, so-called pseudoanonymized data. We have therefore made the transcriptomic and proteomic data available pending relevant permits via https://doi.org/10.48723/cmgs-aj72 This includes a metadata record describing the existing datasets. The analysis code is available at https://github.com/SundlingLab/Malaria_NKcells Data points in graphs derived from pseudoanonymized donors are available from the corresponding author, pending relevant permits. Values for all data points in graphs coming from anonymized donors are reported in the Supporting Data Values.

## Author contributions

PX, PAS, QH, and CS conceptualized the study. PX, PAS, MJL, EB, QH, and CS designed and performed experiments. PX, PAS, MJL, QH, and CS analyzed data. PX, MJL, and CS generated figures and visualizations. AF and BÖ provided scientific input and critical reagents. CS and AF provided funding and student supervision. PX wrote the initial draft with input from CS and MJL. All authors contributed to the revision of the manuscript.

## Conflict of interest

The authors have declared that no conflict of interest exists.

## Funding support

The study was supported by the following grants:

- From Vetenskapsrådet to CS: 2019-0194 and 2023-01943
- From the Åke Wiberg foundation to CS: M18-0076.
- From Magnus Bergvall foundation to CS: 2017-02043 and 2018-02656
- From Karolinska Institutet to CS: FS-2020:0007 and FS-2022:0010
- From Karolinska Institutet to AF: 2019-00992.
- From Vetenskapsrådet to AF for the cohort: 521-2012-3311, 2015-02977, 2018-02688, 2018-04468, 2021-03105
- From Region Stockholm to AF for the cohort: 20150135, 20180409, 960104, 986923
- From Vetenskapsrådet to QH: 2024-02467

## Supplementary Material

Supplemental data

Supplemental video 1

Supplemental video 2

Supplemental video 3

Supporting data values

## Version 1 04/21/2026

In-Press Preview

## Version 2 06/08/2026

Electronic publication
