# Supplementary material for: NK cell cytotoxicity is transiently enhanced during acute malaria and modulated by the host microenvironment
Source: JCI Insight. 2026 Apr 21;11(11):e198687. doi: 10.1172/jci.insight.198687 (PMC13313507; doi:10.1172/jci.insight.198687)
Supplement: Supplemental data [file jciinsight-11-198687-s046.pdf]

Supplemental material

NK cell cytotoxicity is transiently enhanced during acute malaria and modulated by the host microenvironment

Pengjun Xi[1,2,3], Patrick A. Sandoz[4,5,#], Maximilian Julius Lautenbach[1,2,3,#], Eleni Bilev[6], Björn Önfelt[4,6], Anna Färnert[1,2,3], Quirin Hammer[6,7], Christopher Sundling[1,2,3,*]

[1] Division of Infectious Diseases, Department of Medicine Solna, Stockholm, Sweden.
[2] Department of Infectious Diseases, Karolinska University Hospital, Stockholm, Sweden.
[3] Center for Molecular Medicine, Karolinska University Hospital, Stockholm, Sweden.
[4] Department of Applied Physics, Science for Life Laboratory, KTH Royal Institute of Technology, Stockholm, Sweden.
[5] Department of Materials Science and Engineering, Science for Life Laboratory, Uppsala University, Uppsala, Sweden.
[6] Center for Infectious Medicine, Department of Medicine Huddinge, Karolinska Institutet, Stockholm, Sweden.
[7] Institute of Immunology, Christian-Albrecht-University of Kiel, Kiel, Germany.

[#]Equal contribution

File summary

Supplemental Table 1. Clinical table of study participants
Supplemental Table 2. Proteins included in GO terms
Supplemental Table 3. Antibodies and reagents' information

Supplemental Figure 1. Gating strategy for NK cell subsets and counts per microliter
Supplemental Figure 2. Comparison between primary infected and previously exposed donors
Supplemental Figure 3. Pictures of NK cells at different stages in the killing assay
Supplemental Figure 4. NK cell killing assay sorting strategy and repeat experiment
Supplemental Figure 5. Recombinant cytokine stimulation assay
Supplemental Figure 6. IgG depletion of pooled malaria plasma
Supplemental Figure 7. Geometric MFI for malaria plasma effect on NK cells

Supplemental Video 1. GrzB-mediated killing (Red)
Supplemental Video 2. Death Ligand–mediated killing (Green)
Supplemental Video 3. Contact types

Supplemental Table 1. Clinical table of study participants

	Flow cytometry Figure 1 (n=14)	Confocal imaging (n=6)	Previously exposed (n=48)	Primary infected (n=24)
**Age (years)**				
Mean (SD)	34.5 (11.6)	38.8 (21.7)	40.8 (8.9)	36.5 (10.8)
Median (Min, Max)	33 (21, 53)	35.5 (18, 75)	39 (27, 63)	33 (20, 60)
**Gender**				
Male, n (%)	12 (85.7)	4 (66.7)	37 (77.1)	18 (75.0)
Female, n (%)	2 (14.3)	2 (33.3)	11 (22.9)	6 (25.0)
**CMV serology status**				
positive	10 (71.4)	2 (33.3)	43 (89.6)	17 (70.8)
negative	2 (7.1)	0	1 (2.1)	7 (29.2)
Unknown	2 (7.1)	4 (66.7)	4 (8.3)	0
**Sickle cell trait**				
AA	10 (71.4)	2 (33.3)	38 (79.2)	23 (95.8)
AS	2 (7.1)	0	6 (12.5)	0
Unknown	2 (7.1)	4 (66.7)	4 (8.3)	1 (4.2)
**Born in non-endemic country, n (%)**	7 (50.0)	1 (17.7)	0 (0.0)	24 (100.0)
**Born in malaria endemic country (%)**	7 (50.0)	5 (83.3)	48 (100.0)	0 (0.0)
**Self-reported symptom onset before acute samples (days)**				
Mean (SD)	3.5 (1.6)	2.6 (0.5)	4.94 (3.47)	4.71 (2.93)
Median (Min, Max)	3 (1,6)	3 (2, 3)	5 (1, 21)	4 (0, 12)
Missing	2	1	1	1
**Cumulative time of residency in malaria endemic area (years)**				
Mean (SD)	17.6 (13)	26 (0)	25.53 (7.02)	0.59 (1.12)
Median (Min, Max)	18 (0, 39)	26 (26, 26)	25 (14, 39)	0 (0, 3)
Missing	2	4	0	0
**Time since permanent residency in malaria endemic area (years)**				
Mean (SD)	12 (9.7)	-	13.39 (11.32)	-
Median (Min, Max)	12 (1, 32)	-	11.5 (0, 46)	-
Missing	2	6	0	0
**Body temperature at admission (^{o}C)**				
Mean (SD)	38.28 (1.20)	37.88 (1.35)	38.21 (1.23)	38.86 (1.29)
Median (Min, Max)	38.4 (37.7, 39.3)	37.55 (37.1, 40)	38.0 (36.2, 40.6)	39 (36.1, 40.5)
Missing	2	1	0	0
**Leukocytes (WBC) at admission (x10^9/L) (normal range 3.8-8.8)**				
Mean (SD)	5.3 (2.06)	5.03 (2.20)	6.26 (5.53)	4.46 (1.60)
Median (Min, Max)	5.5 (1.4, 8.3)	5.7 (2.9, 6.8)	5.25 (1.9, 35.0)	4.2 (2.1, 7.5)
Missing	2	0	2	0

Supplemental Table 2

Gene Ontology pathway ID					
GO:0002717 positive regulation of natural killer cell mediated immunity	GO:0032814 regulation of natural killer cell activation	GO:0045089 positive regulation of innate immune response	GO:0045824 negative regulation of innate immune response	GO:0050728 negative regulation of inflammatory response	GO:0050729 positive regulation of inflammatory response
Gene ID					
NECTIN2	LEP	TNF	TYRO3	TYRO3	CEBPB
HLA-E	GAS6	FADD	LILRB1	GHRL	TSLP
KLRD1	PGLYRP1	MNDA	YTHDF3	LDLR	TNF
CD160	AXL	TYRO3	PARP1	CDH5	IL6
IL12B	IL15RA	NECTIN2	HLA-E	IL2RA	IL6ST
IL18RAP	HLA-E	PTPRS	TRIM21	CCN3	LGALS1
CRTAM	IL15	BPIFB1	TGFB1	SIRPA	LPL
PVR	KLRD1	LBP	KLRD1	ACP5	LILRA5
SLAMF6	IL12B	CD14	LGALS9	PGLYRP1	LDLR
STAT5B	IL18	FCN2	SERPINB9	SOD1	FABP4
LAG3	FGR	CCL5	HAVCR2	PROC	IL1RL1
	HAVCR2	FCRL3	VSIG4	IL4	PLA2G2A
	STAT5B	IRAK1	GRN	IL13	SERPINE1
		TRIM5	SLAMF8	IL10	LBP
		HLA-E	GFER	MVK	OSMR
		MAP2K6	LYAR	SIGLEC10	IL1B
		IRAK4	MMP12	BCR	IL33
		PIK3AP1	ARG1	CD200R1	IFNG
		HEXIM1	CEACAM1	CD200	HLA-E
		TLR3		CXCL17	IL15
		KLRD1		ADA	OSM
		CLEC7A		HGF	LTA
		CD160		IL12B	TNFSF11
		IL12B		CST7	CCL3
		HSPA1A		TREM2	TLR3
		CD40		FGR	IL16
		TREM2		GSTP1	TNFRSF11A
		COLEC12		CX3CL1	IL12B
		CASP1		PLA2G10	CTSC
		IL18RAP		TNFRSF1B	IL18
		EREG		TNFRSF1A	TREM2
		CRTAM		GRN	CCL24
		LY96		SLAMF8	CASP1
		PVR		FCGR2B	CX3CL1
		ADAM8		YES1	MMP8
		CTSS		LYN	ADAM8
		HAVCR2		NPY	APP
		LILRA2		MDK	IL17RA
		NINJ1		TEK	PLA2G7
		PQBP1		NT5E	TNFRSF1A
		MAVS		FURIN	GRN
		BIRC2		SRC	PARK7
		RIGI			NINJ1
		CLEC6A			CD28
		SLAMF6			STAT5B
		STAT5B			S100A12
		LAG3			CCN4
		ERBIN			MDK
		LYN			
		MMP12			
		CD300LF			
		SRC			

Supplemental Table 3

Antibodies used for figure 1

Antibodies	Clone	Source	Identifier
CD8 BUV737	SK1	BD	Cat# 612754, RRID:AB_2870085
Ki67 BUV395	B56	BD	Cat# 564071, RRID:AB_2738577
CD56 BU786	NCAM16.2	BD	Cat# 564058, RRID:AB_2738569
HLA-DR BV605	G46-6	BD	Cat#562845; RRID:AB_2744478
CD16 BV510	3G8	BD	Cat#563830; RRID:AB_2938676
CD57 eFluor450	TB01	Thermo Scientific	Cat#48-0577-42; RRID:AB_2016680
Perforin PerCPCy5.5	δG9	BD	Cat# 563762, RRID:AB_2738409
FcRy FITC	Polyclonal	Milli-Mark	Cat# FCABS400F; RRID:AB_11203492
CD3 PE-Cy7	UCHT1	BD	Cat# 563423, RRID:AB_2738196
CD14 PE-Cy7	M5E2	BD	Cat# 557742, RRID:AB_396848
CD19 PE-Cy7	SJ25C1	BD	Cat# 557835, RRID:AB_396893
PLZF PE-CF594	R17-809	BD	Cat# 565738, RRID:AB_2739339
NKG2C PE	S19005E	Biolegend	Cat#375004; RRID:AB_2888871
CD38 APC-Fire810	HB-7	Biolegend	Cat#356644; RRID:AB_2860936
NKG2A APC-Fire750	S19004C	Biolegend	Cat#375116; RRID:AB_2888866
CD7 R718	4H9	BD	Cat# 568384, RRID:AB_3684232
Granzyme B APC	QA16A02	Biolegend	Cat# 372204, RRID:AB_2687028
CountBright absolute counting beads		Thermo Scientific	Cat#C36950

Antibodies and reagents used for figure 5 and 6

Antibodies	Clone	Source	Identifier
Viability dye aqua		Thermo Scientific	Cat#L34957;
CD57, PacificBlue	HNK-1	BioLegend	Cat#359608; RRID:AB_2562459
CD19, BV570	HIB19	BioLegend	Cat#302236; RRID:AB_2563606
CD3, PE-Cy5	UCHT1	BioLegend	Cat#300410; RRID:AB_314064
NKG2C, PE	REA205	Miltenyi	Cat#130-119-776; RRID:AB_2751835
NKG2A, PE-Vio770	REA110	Miltenyi	Cat#130-113-567; RRID:AB_2726172
CD56, BUV737	NCAM16.2	BD	Cat#612767; RRID:AB_2860005
CD14, BV605	M5E2	BioLegend	Cat#301834; RRID:AB_2563798
CD16, BV785	3G8	BioLegend	Cat#302046; RRID:AB_2563803
Granzyme B, AlexaFluor700	GB11	BD	Cat#560213; RRID:AB_1645453
Perforin, FITC	dG9	BioLegend	Cat#308104; RRID:AB_314701
IL-1b		PeproTech	Cat#200-01B-10UG
IL-12		BioLegend	Cat#573002
IL-15		BioLegend	Cat#570316
IL-18	E.coli-derived	R&D System	Cat#9124-IL/CF
TGF-b1		PeproTech	Cat#100-21-100G
IgG1 Isotype Antibody	11711	R&D System	Cat#MAB002; RRID:AB_357344

anti-TGF-b	1D11	R&D System	Cat#MAB1835; RRID:AB_357931
anti-IL12 p70	24910	R&D System	Cat#MAB219; RRID:AB_2123616
anti-IL15	34593	R&D System	Cat#MAB247; RRID:AB_2124578

Reagents used for confirmation of IgG antibody depletion

	Clone	Source	Identifier
Magnetic COOH beads (region 26)		**Bio-Rad**	**Cat#MC10026-01**
Magnetic COOH beads (region 27)		Bio-Rad	Cat#MC10027-01
Magnetic COOH beads (region 35)		Bio-Rad	Cat#MC10035-01
Magnetic COOH beads (region 44)		Bio-Rad	Cat#MC10044-01
Magnetic COOH beads (region 52)		Bio-Rad	Cat#MC10052-01
Magnetic COOH beads (region 62)		Bio-Rad	Cat#MC10062-01
Zeba spin desalting columns		Thermo Scientific	Cat#89882
Bio-Plex Amine coupling kit		Bio-Rad	Cat#171-406001
EDAC		Bio-Rad	Cat#153-0990
Sulfo-NHS		Thermo Scientific	Cat#24510
96-well microtiter plates		Bio-Rad	Cat#171025001
PE-conjugated Goat Anti-Human IgG		Jackson ImmunoResearch Laboratories	Cat#109-116-170; RRID:AB_2337681
Mouse anti-human IgG1 Hinge-PE	4E3	SouthernBiotech	Cat#9052-09; RRID:AB_2796621
Mouse anti-human IgG3 Hinge-PE	HP6050	SouthernBiotech	Cat#9210-09; RRID:AB_2796701
Mouse anti-human IgM Hinge-PE	SA-DA4	SouthernBiotech	Cat#9020-09; RRID:AB_2796577
Ab SpinTrap		Cytiva	Cat#28403

Antibodies for immune cell subset profiling (Supplemental figure 2)

Antibodies	Clone	Source	Identifier
CD14, BB700	MφP9	BD	Cat# 566465; RRID:AB_2739737
CD57, BB515	NK-1	BD	Cat#565285; RRID:AB_2739155
gd TCR, PE-Cy7	11F2	BD	Cat#655410; RRID:AB_2870377
CD38, PE-Cy5	HIT2	BD	Cat#555461; RRID:AB_395854
CD25, PE-CF594	M-A25	BD	Cat#562403; RRID:AB_11151919
Vd2, PE	b6	BD	Cat#555739; RRID:AB_396082
CD3, APC-H7	SK7	BD	Cat#560176; RRID:AB_1645475
CD16, Alexa Fluor 700	3G8	BD	Cat#560713; RRID:AB_1727430
CCR7, Alexa Fluor 647	3D12	BD	Cat#557734; RRID:AB_396842
CD56, BV786	NCAM16.2	BD	Cat#564058; RRID:AB_2646835
CD19, BV711	SJ25C1	BD	Cat#563036; RRID:AB_2737968
CD45RA, BV650	HI100	BD	Cat#563963; RRID:AB_2738514
HLA-DR, BV605	G46-6	BD	Cat#562845; RRID:AB_2744478
Aqua LIVE/DEAD		Thermo Scientific	Cat#L34966

CD127, BV421	HIL-7R-M21	BD	Cat#562436; RRID:AB_11151911
CD8, BUV737	SK1	BD	Cat#612754; RRID:AB_2870085
CD4, BUV395	SK3	BD	Cat#563550; RRID:AB_2738273

Supplemental Figure 1

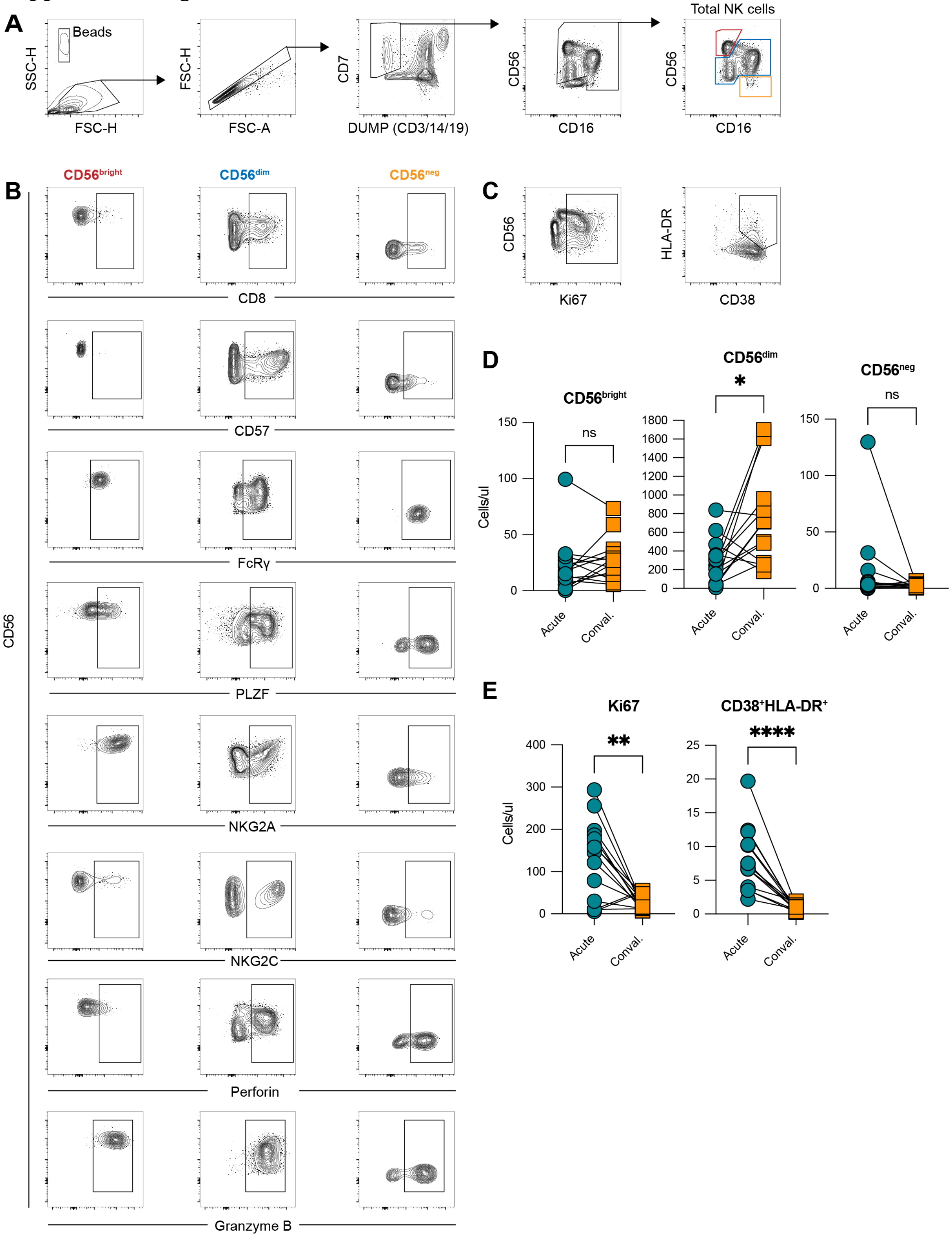

Supplemental Figure 1. Gating strategy and cell counts*. (A) Gates identifying added cell counting beads and NK cell subsets. (**B**) Gates for indicated protein markers among CD56bright (left), CD56dim (middle), and CD56neg (right) NK cells. (**C**) Gates for proliferation (Ki67) and activation (CD38 versus HLA-DR). (**D**) Cells per μL calculated using countbright beads for NK cell subsets (n=14). **(E)** Cells per μL of proliferating and activated total NK cells (n=14). P-values were calculated between acute and convalescent (12 month) time-points using two-tailed paired students t-tests. Significance is*

*indicated as: ns = not significant, p>0.05. A p-value < 0.05 was considered statistically significant with *p<0.05, **p<0.01, ****p<0.0001.*

Supplemental Figure 2

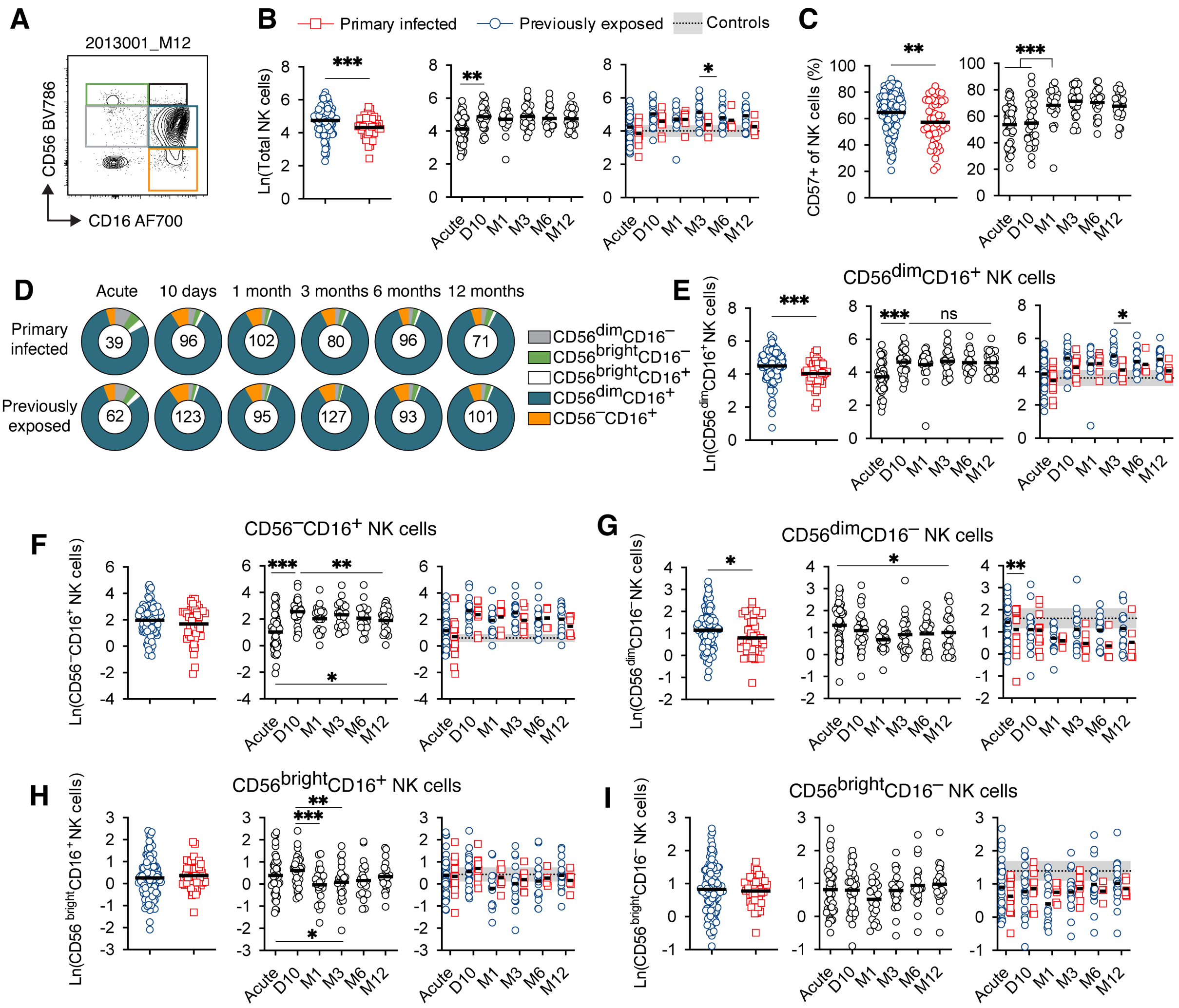

***Supplemental Figure 2*. Comparison between primary infected and previously exposed donors following acute malaria.** *(**A**) Representative FACS plot showing gating strategy for NK cell subsets based on CD56 and CD16 expression. (**B**) Ln Total NK cell counts per 1000 live cells. Left: Comparison between primary-infected (red, n = 50 samples) and previously exposed individuals (blue, n = 115 samples) across all time points. Middle: Longitudinal analysis of total NK cell counts (groups combined; n = 24-45 samples per time point). Right: Time-course analysis stratified by exposure history. (**C**) Frequency of CD57⁺ NK cells. Left: Combined data across all time points. Right: Longitudinal analysis by exposure group. (**D**) Proportional contribution of each NK cell subset to the total NK population at each time point and exposure group. Numbers indicate total NK cells per 1000 live cells. (**E–I**) Longitudinal analysis of individual NK cell subsets (indicating Ln cell number per 1000 live cells): (**E**) $CD56^{dim}CD16^{+}$ NK cells, (**F**) $CD56^{-}CD16^{+}$ NK cells, (**G**) $CD56^{dim}CD16^{-}$ NK cells, (**H**) $CD56^{bright}CD16^{+}$ NK cells, and (**I**) $CD56^{bright}CD16^{-}$ NK cells. Statistical analyses were performed using unpaired Welch's two-tailed t-test, or mixed-effects analyses followed by two-tailed t-tests with correction for multiple testing. Only significant comparisons are shown: A p-value < 0.05 was considered statistically significant with *p < 0.05, **p < 0.01, ***p < 0.001, ****p < 0.0001. Symbols indicate individual donors. The dotted line indicates the mean NK cell count in healthy Swedish donors (n = 10), with the shaded area representing the 95% confidence interval.*

Supplemental Figure 3

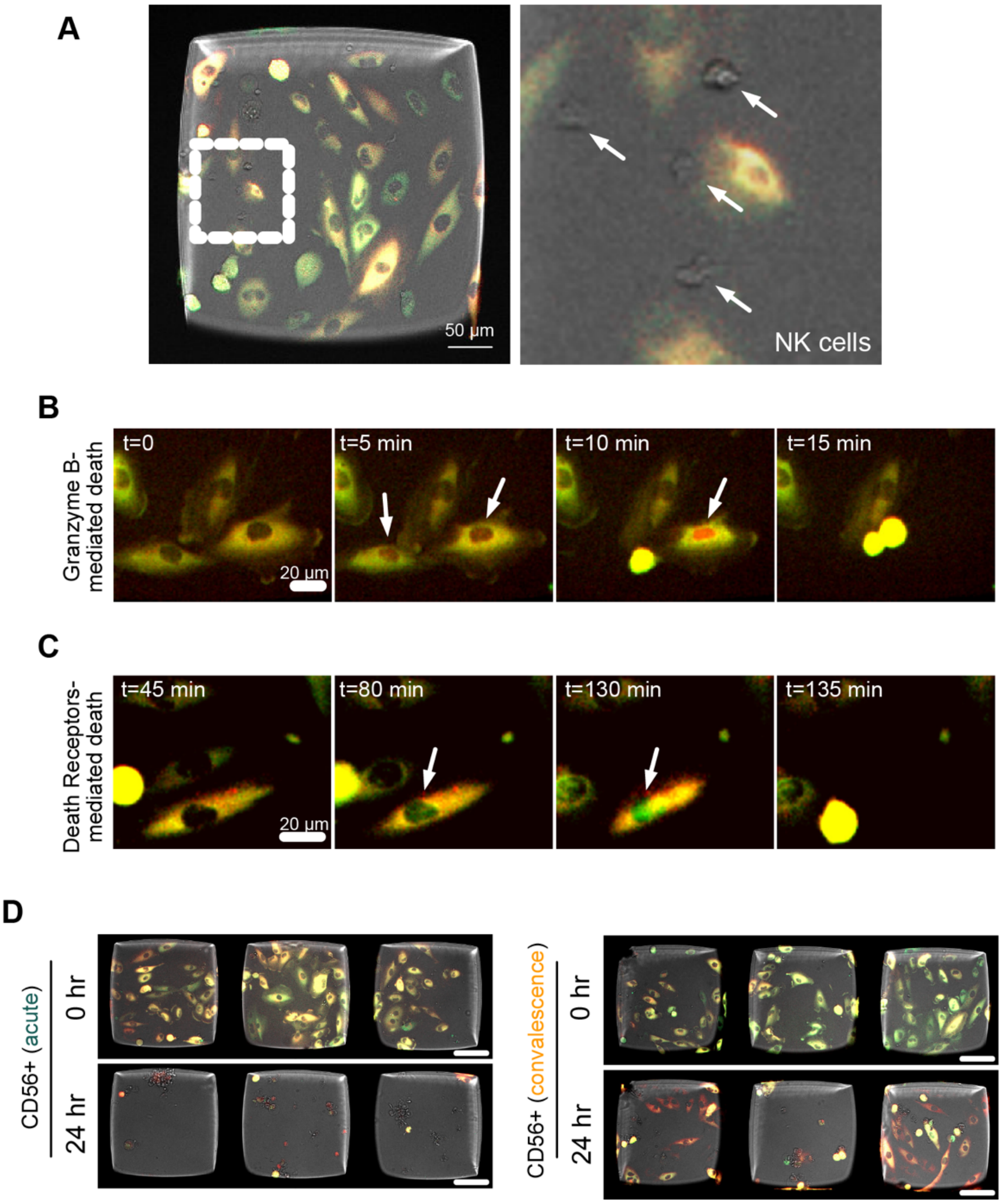

Supplemental Figure 3. Zoom-in view of confocal microscopy time-lapse killing assay. *(A) A zoom-in view of NK cells (white arrows) and target cells (green/yellow). The left panel shows a microwell with cells. The right panel shows a zoom-in view where NK cells which are unlabeled and transparent, are indicated by arrows. (**B**) Zoom-in view of how target cells are killed by NK cell using granzyme B (arrows), indicated by the colour changing from unstained to red (mCherry expressed) with time. (**C**) Zoom-in view of a target cell that is killed by NK cell with death ligands (white arrow), indicated by the colour changing from unstained to green (GFP expressed) with time. (**D**) A screenshot of three microwells at co-culture initiation (0 hours) and at 24 hours of the experiment. This illustrates the enhanced killing efficiency of acute compared with convalescent NK cells, where target cells remain after 24 hours.*

Supplemental Figure 4

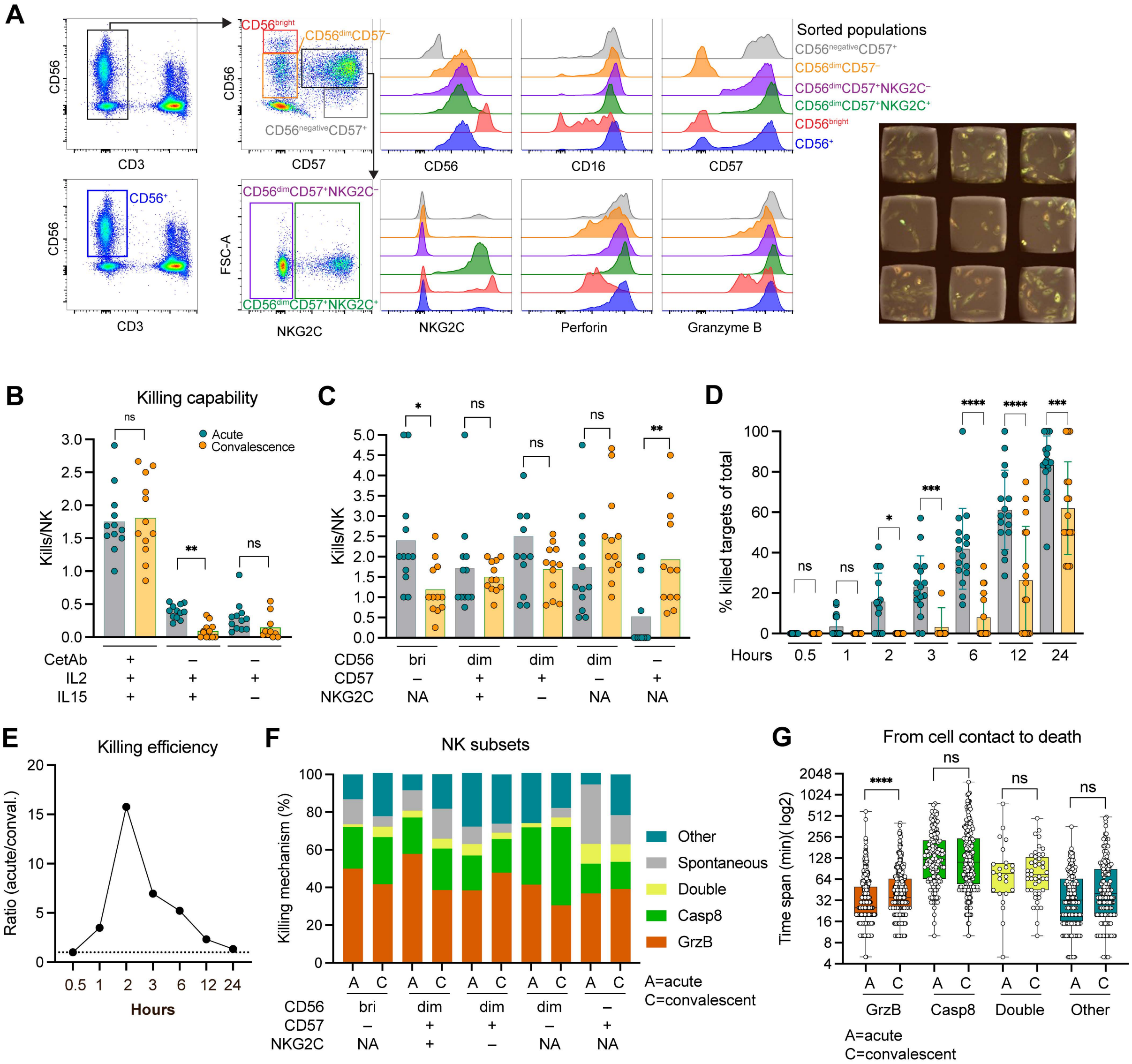

Supplemental Figure 4. NK cell killing assay sorting strategy and repeat experiment. *(A) Gating strategy to sort total CD56^{+} (blue), CD56^{bright}CD57$^{–}$ (red), CD56^{dim}CD57$^{–}$ (orange), CD56^{dim}CD57^{+}NKG2C^{-} (purple), CD56^{dim}CD57^{+}NKG2C^{+} (green), and CD56^{-}CD57^{+} (grey) NK cells. Middle panel: Histograms indicate protein expression of the indicated markers. Right panel: Representative picture of microwells containing target cells and NK cells. (**B**) Total CD56^{+} NK cell killing capacity when cultured with target cells in the presence or absence of cetuximab (CET), IL15, and IL2 over 2 days (n=12 microwells). (**C**) Killing capacity of NK cell subsets in the presence of cetuximab antibody, IL15, and IL2 (n=12 microwells). NA indicates that the marker was not assessed during cell sorting. (**D**) The percentage of target cells killed at different time-points. NK cells from acute malaria in blue and convalescence in orange (n=12 microwells). (**E**) Killing efficiency calculated as the ratio between kills/NK for acute over convalescence at different time-points. (**F**) Total CD56^{+} NK cell mediated killing mechanism for different NK cell subsets. GrzB indicate granzyme B-mediated and Casp8 indicate caspase 8-mediated. (**G**) The time in minutes of contact between an NK cell and their target cells associated with the different killing mechanisms. Each dot represents a killing event (n=24 – 451 killing events). Statistical analyses were done for B and C by two-tailed unpaired Student's t-tests, and statistical analyses for (D) were done using one-way ANOVA, with ns = p>0.05. A p-value < 0.05 was considered statistically significant with *p<0.05, **p<0.01, ***p<0.001, ****p<0.0001. (B-H) The second representative donor based on 12 microwells.*

Supplemental Figure 5

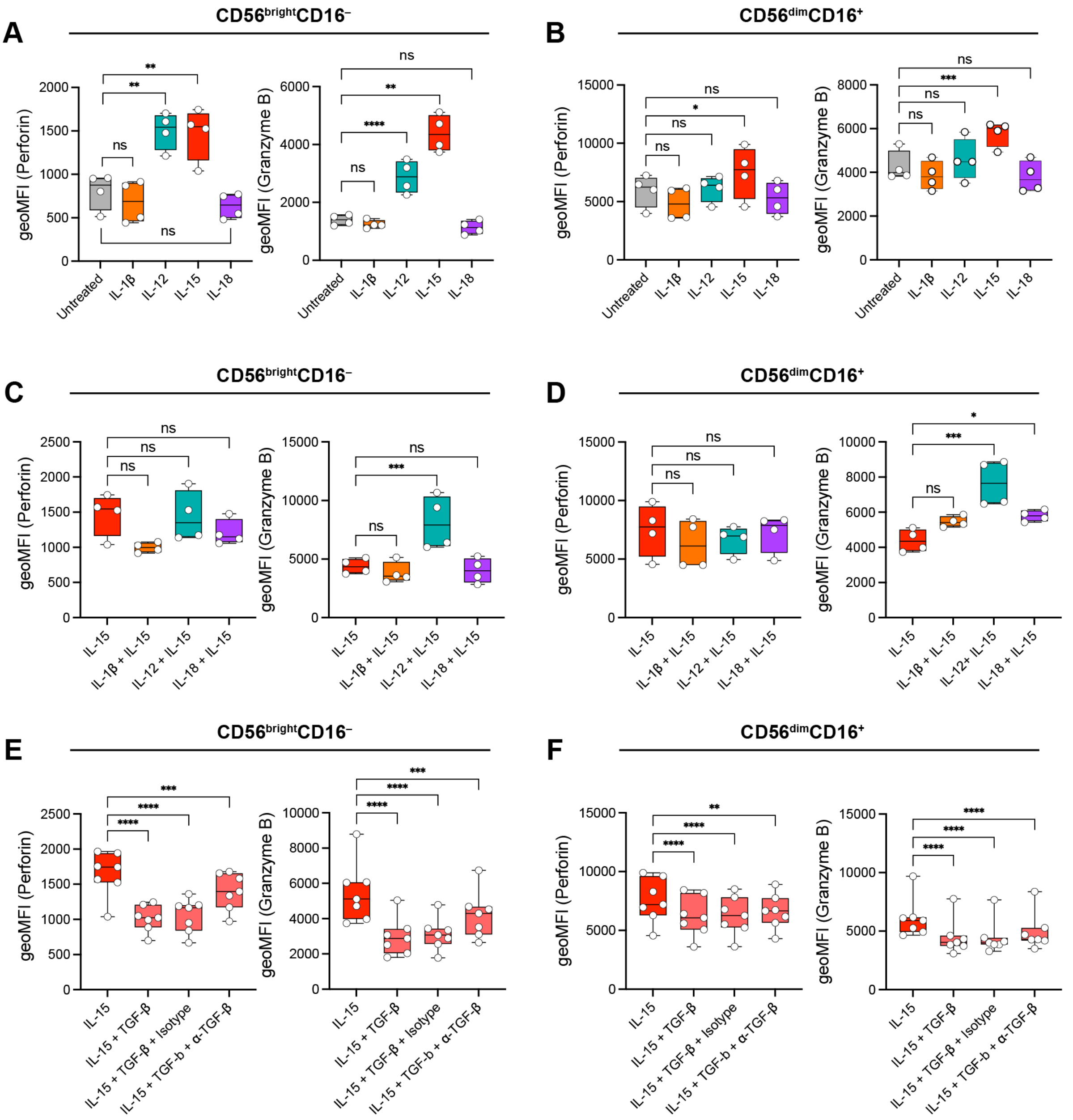

***Supplemental Figure 5. Geometric mean fluorescent intensity for in vitro cytokine stimulation of isolated NK cells from healthy PBMC**. (**A-B**) Intracellular expression of perforin (left) and granzyme B (right) in (**A**) $CD56^{bright}CD16^{-}$ and (**B**) $CD56^{dim}CD16^{+}$ NK cells following 24h left untreated (grey) or stimulated with IL-1β (orange), IL-12 (turquoise), IL-15 (red), or IL-18 (purple). Values correspond to geometric mean fluorescent intensity. (**C-D**) Perforin (left) and granzyme B (right) expression in (**C**) $CD56^{bright}CD16^{-}$ and (**D**) $CD56^{dim}CD16^{+}$ NK cells following combined cytokine stimulation. (**E-F**) Perforin (left) and granzyme B (right) expression in (**E**) $CD56^{bright}CD16^{-}$ and (**F**) $CD56^{dim}CD16^{+}$ NK cells after stimulation with IL-15 alone, IL-15 + TGF-β, IL-15 + isotype control, or IL-15 + anti–TGF-β antibody. Statistical analyses were done using repeated measures one-way ANOVA with ns=$p > 0.05$. A p-value < 0.05 was considered statistically significant with *$p < 0.05$, **$p < 0.01$, ***$p < 0.001$, ****$p < 0.0001$. Each dot represents one donor (n=4-7) with data pooled from three experiments. Results were pooled from three separate experiments including in total 4-7 donors.*

Supplemental Figure 6

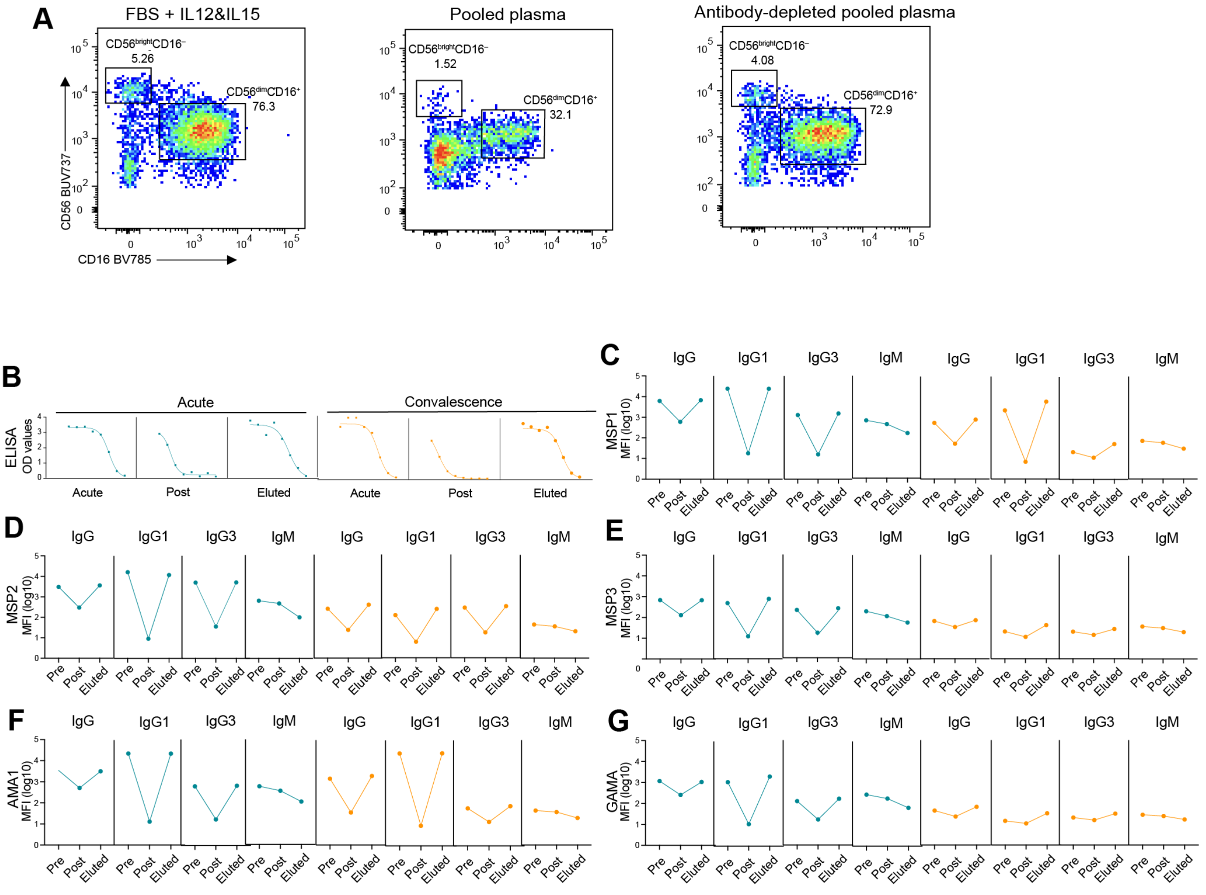

Supplemental Figure 6. IgG depletion of pooled malaria plasma. *(**A**) The gating strategy for in vitro stimulated CD56^{bright}CD16^{-}and CD56^{dim}CD16^{+} NK cells. Left FACS plot indicates NK cells cultured with fetal bovine serum supplemented with IL-12+IL-15, middle panel demonstrates NK cells cultured with 20% pooled plasma (containing IgG), the right panel demonstrates NK cells cultured with 20% pooled plasma where the IgG has been removed. (**B**) ELISA for total IgG antibodies before (pre) and after (post) removal of IgG from the pooled acute (blue) and convalescent (orange) plasma respectively. (**C-G**) Multiplex bead-based luminex assay to validate total IgG, IgG1, IgG3 and IgM antibody levels in the pooled acute (blue) and convalescent (orange) plasmas, respectively. This has done to test with five merozoites antigens, (C) MSP1-19, (D) MSP2, (E) MSP3, (F) AMA1 and (G) GAMA. All antigens were derived from P. falciparum.*

Supplemental Figure 7

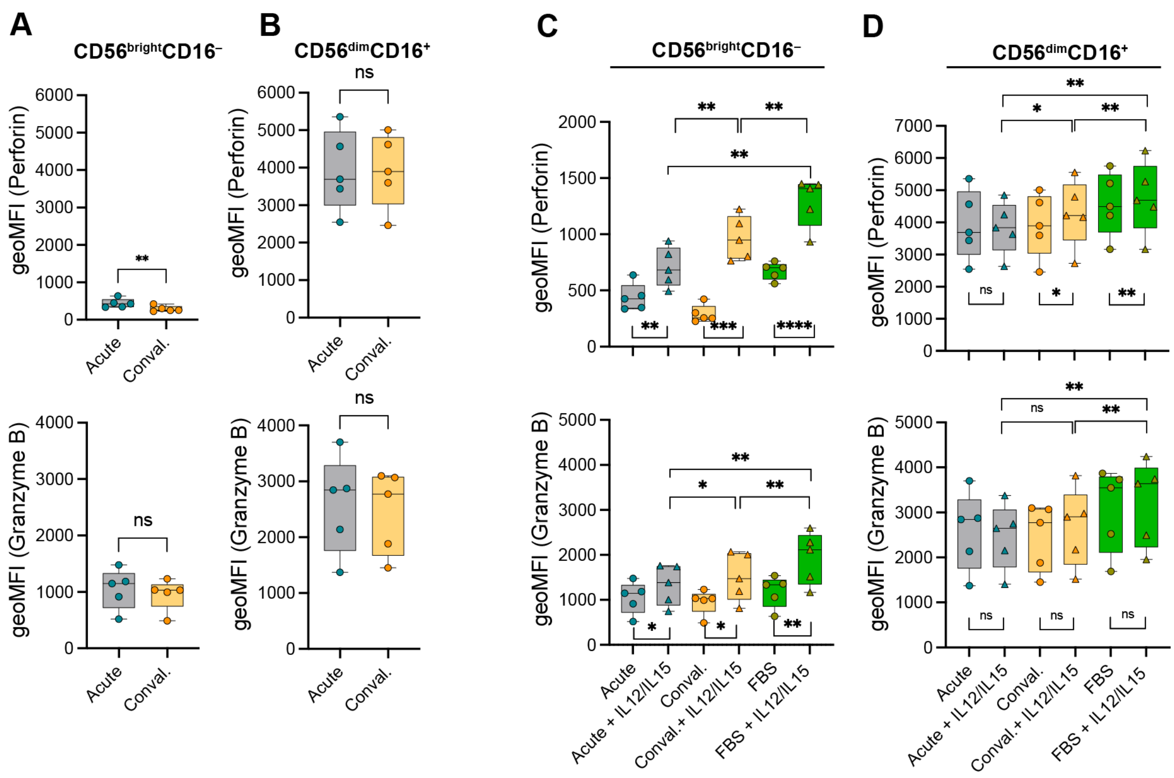

***Supplemental Figure 7. Geometric mean fluorescent intensity for malaria plasma effect on purified NK cells**. (**A**) Intracellular expression of perforin (top panel) and granzyme B (bottom panel) in (**A**) CD56^{bright}CD16^{-} or (**B**) CD56^{dim}CD16^{+} NK cells cultured with 20% IgG-depleted pooled plasma from acute (blue) or convalescent (orange) malaria patients. Values correspond to geometric mean fluorescent intensity of the gated population. (**C-D**) Geometric mean fluorescent intensity for perforin (top) and granzyme B (bottom) expression in (**C**) CD56^{bright}CD16^{-} or (**D**) CD56^{dim}CD16^{+} NK cells cultured with pooled acute or convalescent plasma or fetal bovine serum (FBS), with or without additional IL-12 and IL-15. Values correspond to geometric mean fluorescent intensity of the gated population. Statistical analyses were performed using two-tailed paired Student's t-tests. Significance is indicated as: ns = not significant. A p-value < 0.05 was considered statistically significant with *p < 0.05, **p < 0.01, ***p < 0.001, ****p < 0.0001. Experiments were done on cells sorted from 5 separate donors.*

Description of Additional Supplemental video Files

File Name: Supplemental Video 1
Description: Example of granzyme B-mediated killing of A498^{GBDR} target cells. Target cells stably express the dual fluorescent reporter NES-ELQTD-GFP-T2A-NES-VGPD-mCherry co-cultured with unlabelled NK cells. Left panel shows the fluorescence of A498^{GBDR} cells with mixed GFP (green) and mCherry (red) cytoplasm indicated in yellow. When under attack of an NK cell, the fluorescent reporter is cleaved and mCherry (granzyme B-mediated death) can diffuse into the nucleus, making the nucleus turnsfrom unstained to red. The middle panel is the brightfield of the granzyme B mediated killing, showing small fast moving NK cells. The right panel shows the fluorescence and brightfield merged views of granzyme B-mediated killing.

File Name: Supplemental Video 2
Description: Example of death ligand-mediated killing of A498^{GBDR} target cells. Target cells stably express the dual fluorescent reporter NES-ELQTD-GFP-T2A-NES-VGPD-mCherry co-cultured with unlabelled NK cells. Left panel shows the fluorescence of A498^{GBDR} cells with mixed GFP (green) and mCherry (red) cytoplasm indicated in yellow. When under attack of an NK cell, the fluorescent reporter is cleaved and GFP (death ligand-mediated death) can diffuse into the nucleus, making the nucleus turn from unstained to green. The middle panel is the brightfield of the death ligand-mediated killing, showing small fast moving NK cells. The right panel shows the fluorescence and brightfield merged views of death ligand-mediated killing.

File Name: Supplemental Video 3
Description: Example of committed contact types of how NK cells contact A498^{GBDR} target cells. A498^{GBDR} are relatively large with yellow cytoplasm and NK cells are relatively small unlabelled (transparent) cells. Within the video, **A** indicates the migration of an NK cell, where it only has one contact with the target cell. **B** (stable) shows the committed contact, defined as NK cells having contact with target cells more than 2 frames (where 2 frames is approximately 10 min). **C** shows the anchor point contact time where the NK cells (arrow pointed) shaped like an anchor to contact target cells.
